# Long-term evolution of *Streptococcus mitis* and *Streptococcus pneumoniae* leads to higher genetic diversity within rather than between human populations

**DOI:** 10.1371/journal.pgen.1011317

**Published:** 2024-06-06

**Authors:** Charlotte Davison, Sam Tallman, Megan de Ste-Croix, Martin Antonio, Marco R. Oggioni, Brenda Kwambana-Adams, Fabian Freund, Sandra Beleza

**Affiliations:** 1 Department of Genetics and Genome Biology, University of Leicester, Leicester, United Kingdom; 2 Medical Research Council Unit The Gambia at the London School of Hygiene & Tropical Medicine, Fajara, The Gambia; 3 Centre for Epidemic Preparedness and Response, London School of Hygiene & Tropical Medicine, London, United Kingdom; 4 Department of Infection Biology, Faculty of Infectious and Tropical Diseases, London School of Hygiene & Tropical Medicine, London, United Kingdom; 5 Department of Pharmacy and Biotechnology, University of Bologna, Bologna, Italy; 6 Department of Clinical Sciences, Liverpool School of Tropical Medicine, Liverpool, United Kingdom; 7 Malawi Liverpool Welcome Programme, Blantyre, Malawi; 8 Division of Infection and Immunity, University College London, London, United Kingdom; University of Warwick, UNITED KINGDOM

## Abstract

Evaluation of the apportionment of genetic diversity of human bacterial commensals within and between human populations is an important step in the characterization of their evolutionary potential. Recent studies showed a correlation between the genomic diversity of human commensal strains and that of their host, but the strength of this correlation and of the geographic structure among human populations is a matter of debate. Here, we studied the genomic diversity and evolution of the phylogenetically related oro-nasopharyngeal healthy-carriage *Streptococcus mitis* and *Streptococcus pneumoniae*, whose lifestyles range from stricter commensalism to high pathogenic potential. A total of 119 *S*. *mitis* genomes showed higher within- and among-host variation than 810 *S*. *pneumoniae* genomes in European, East Asian and African populations. Summary statistics of the site-frequency spectrum for synonymous and non-synonymous variation and ABC modelling showed this difference to be due to higher ancestral bacterial population effective size (*N*_*e*_) in *S*. *mitis*, whose genomic variation has been maintained close to mutation-drift equilibrium across (at least many) generations, whereas *S*. *pneumoniae* has been expanding from a smaller ancestral bacterial population. Strikingly, both species show limited differentiation among human populations. As genetic differentiation is inversely proportional to the product of effective population size and migration rate (*N*_e_*m)*, we argue that large *N*_*e*_ have led to similar differentiation patterns, even if *m* is very low for *S*. *mitis*. We conclude that more diversity within than among human populations and limited population differentiation must be common features of the human microbiome due to large *N*_*e*_.

## Author summary

The genetic variation of human-associated bacteria and the evolutionary mechanisms leading to that variation are crucial for the establishment of highly contextual interactions with their host, giving rise to phenotypes such as virulence. Here, we studied *Streptococcus mitis* and *Streptococcus pneumoniae*, who share a common ancestor but evolved lifestyles on different ends of the pathogenic potential spectrum: *S*. *mitis* is mainly commensal, whereas *S*. *pneumoniae* has high pathogenic potential. Genomic variation of worldwide healthy-carriage strains is considerably higher for *S*. *mitis*. We show this to be due to a larger ancestral population in *S*. *mitis*, whose population effective size (*N*_*e*_) and mutation gain and loss (by genetic drift) have been kept stable over the generations. In contrast, *S*. *pneumoniae* population has expanded from an ancestral population with smaller *N*_*e*_. We also observe low genetic differentiation among populations in both species. We deduce that this is due to large *N*_*e*_ which, even with limited dispersal rate as in *S*. *mitis*, leads to significant effective dispersal among populations. As both species’ properties of *N*_*e*_ and dispersal are common among human-associated bacteria, more diversity within rather than among human populations and limited population differentiation must be common features of the human microbiome.

## Introduction

There is evidence that healthy carriage of human-associated bacteria have coevolved with humans over thousands of years [1–3], and that this has generated significant bacterial genetic diversity among hosts [4]. Reconstructions of within-species single nucleotide variants (SNVs) from metagenomic samples from the gut showed that bacterial strain diversity in human populations is globally consistent with theoretical expectations of long-term evolution by stochastic fluctuation of allele frequencies over the generations (genetic drift) and purifying selection [4,5]. However, these studies have also uncovered a wide range of genetic variation among species. This is reflective of independent evolutionary histories enabling bacteria with different ecological trajectories. Importantly, these evolutionary histories have resulted from interactions with humans that range from bacteria with a stricter commensal lifestyle to others exhibiting high pathogenic potential. Characterization of the genetic variation of human-associated bacterial species on different ends of the pathogenic potential spectrum and the evolutionary mechanisms leading to that variation is, therefore, crucial for understanding bacterial adaptation to their host environments, as well as the evolution of phenotypes such as virulence.

An interesting case of phylogenetic related species that show contrasting interactions with humans are the two upper respiratory tract inhabitants *Streptococcus mitis* and *Streptococcus pneumoniae*. *S*. *mitis* is one of the most prevalent species of the oropharyngeal microbiome [6,7], where it resides as a commensal. Because of its abundance at birth and throughout life, *S*. *mitis* is an excellent model for comprehensive analysis of variation and diversification of the oral microbiome. Compositional similarity between mother and child indicates vertical (familial) transmission of the oral microbiome [8]. However, early investigations into genetic diversity of *S*. *mitis*, although impeded by difficulties in distinguishing related species within the Mitis phylogenetic group [3,9], found multiple genotypes in new-born infants both matching and not matching parental genotypes, suggesting some degree of horizontal transmission of the Mitis group including *S*. *mitis* [10,11]. This is corroborated by recent oral metagenome studies that discovered a median sharing of 32% of oral strains between cohabitating individuals, and 3% between non-cohabiting individuals in the same population [12]. High *S*. *mitis* diversity within and between hosts is supported by analyses of the highly differentiating *gdh* gene, of a combination of multi-locus sequence alignment (MLSA) genes, and of a small number of genomes [13–15]. These patterns were associated with their lower rate of transmission between unrelated and non-cohabitating individuals leading to lineage isolation and, consequently, to preclusion of potential homogenization of the mitis gene pool due to homologous recombination [14,15]. According with this view, discrete clusters of genetic variation tracking host genomic variation across populations should be observed, for instance in a similar manner to the stomach-colonizer and vertically transmitted *Helicobacter pylori* [16]. Recent studies have shown this to be the case for gut microbiome species [17], although the correlation between host and commensal genetic diversities seems to be weak [18–20]. The apportionment of genetic diversity of human commensals within and between human populations is still a subject of debate, and the factors that contribute to bacterial population differentiation need further consideration. This is important because the amount of population variation within and among populations contribute to the evolutionary potential of a species.

On the other hand, *S*. *pneumoniae* is a frequent colonizer of the nasopharynx that is associated with high human mortality and morbidity worldwide [21]. Although nasopharyngeal colonization is usually asymptomatic, it is considered an essential step preceding invasive and non-invasive pneumococcal disease [22,23]. In addition, carriage serves as a source of pneumococci that can be transmitted within and between households. The rate of horizontal transmission between households is known to be high in *S*. *pneumoniae* [24,25], which will influence the level and distribution of genetic variation in the overall population of *S*. *pneumoniae* across host populations. Therefore, pneumococcal carriage is a key stage in the evolution of this organism. However, *S*. *pneumoniae* population genetic variation has been mostly characterised in relation to invasive disease [26–34] or in the context of changes after vaccine introduction [35–43], and few recent studies address the genetic variation of carriage *S*. *pneumoniae* within a population [44–49].

Differences in transmission rates between *S*. *mitis* and carriage *S*. *pneumoniae* (considerably higher for *S*. *pneumoniae*) are expected to lead to more geographic isolation of the former in comparison to the latter, and this is expected to lead to the observation of higher geographic structure in *S*. *mitis* than in *S*. *pneumoniae*. However, under the standard neutral model, genetic differentiation among populations is inversely proportional to the product of effective population size and migration rate (*N*_e_*m*; [50]). This means that in populations with large *N*_*e*_, even in cases where *m* is minimal, effective migration will overcome genetic drift (proportional to 1/*N*_*e*_) leading to low long-term population differentiation.

Here, we sought to gain a better understanding about the evolution and population differentiation of healthy carriage of *S*. *mitis* and *S*. *pneumoniae* across human populations. We collected bacterial samples from European, East Asian and African hosts, which compose the major divisions of human genetic diversity [51], in this way, sampling across deeper evolutionary time scales. We observe that the apportionment of genetic diversity is higher within populations than between populations, and that this is related with the large *N*_*e*_ for both species. However, genomic variation is considerable higher for *S*. *mitis* due to a bigger ancestral bacterial population that has been maintained close to mutation-drift equilibrium across (at least many) generations; in *S*. *pneumoniae*, contemporary diversity is inferred to be due to a population expansion from an ancestral population with smaller *N*_*e*_. Both species have been evolving under purifying selection, but there are signatures of diversifying selection which have not led to high intermediate-frequency alleles.

## Results

### *S*. *mitis* has considerably higher within-host diversity than carriage *S*. *pneumoniae*

We collected a representation of within- and between-host genomic variation of healthy colonisation of *S*. *mitis* and *S*. *pneumoniae* across major human population groups. The *S*. *mitis* dataset consists of a total of 101 newly collected and 18 publicly available isolates from 46 independent hosts from Africa (n = 17 hosts/32 isolates), East Asia (n = 6 hosts/37 isolates), and Europe (n = 24 hosts/50 isolates). More than one isolate was collected from 18 hosts, ranging from 2 to 10 isolates per host. The African dataset included 20 isolates from five pairs of related individuals (two mother-child pairs, two sibling pairs and one sibling trio), whereas the European and East Asian isolates were obtained from unrelated individuals only.

To obtain a sample of the true general diversity of carriage *S*. *pneumoniae* populations that is not biased by the action of strong recent selection, the *S*. *pneumoniae* dataset was obtained from published pre-vaccination studies [42,44,46] and comprises a total of 810 isolates from asymptomatic hosts (carriage isolates) from Africa (n = 90 hosts /230 isolates), East Asia (n = 480 hosts/isolates) and Europe (n = 100 hosts/isolates). The African dataset included within-host genomic variation, with the number of isolates collected from 71 hosts ranging from two to five. In this dataset, the total number of serotypes, sequence types and Global Pneumococcal Sequence Clusters (GPSCs) of shared evolutionary history as previously defined [52,53] is 63, 253 and 135.

Genetic diversity and presence of clonal relationships within the host, within families and between hosts were assessed for both species by calculating the number of SNV differences between every pair of isolates in the total dataset and within each of those three levels of host relatedness. For *S*. *mitis*, the mean number of pairwise SNV differences in the total sample is 30,190 (Interquartile range, IQR: 29,050–32,580). We observed clonal relationships in ~one percent of total pairwise comparisons (<1000 SNVs), mostly from within-the host, but also between related individuals (two sibling pairs) and between unrelated individuals’ isolates collected in the same geographic region (five pairs; S1A Fig). Pairwise SNV comparisons within the host (mean = 24,366; IQR: 25,298–30,358) and between related hosts (mean = 25,411; IQR: 24,822–32,769) are slightly smaller than pairwise SNV comparisons between unrelated hosts (mean = 30,531; IQR: 29,189–38,379) (Kruskal-Wallis test, p-value≤ 7.08x10^-5^; S1C Fig).

**Fig 1:**
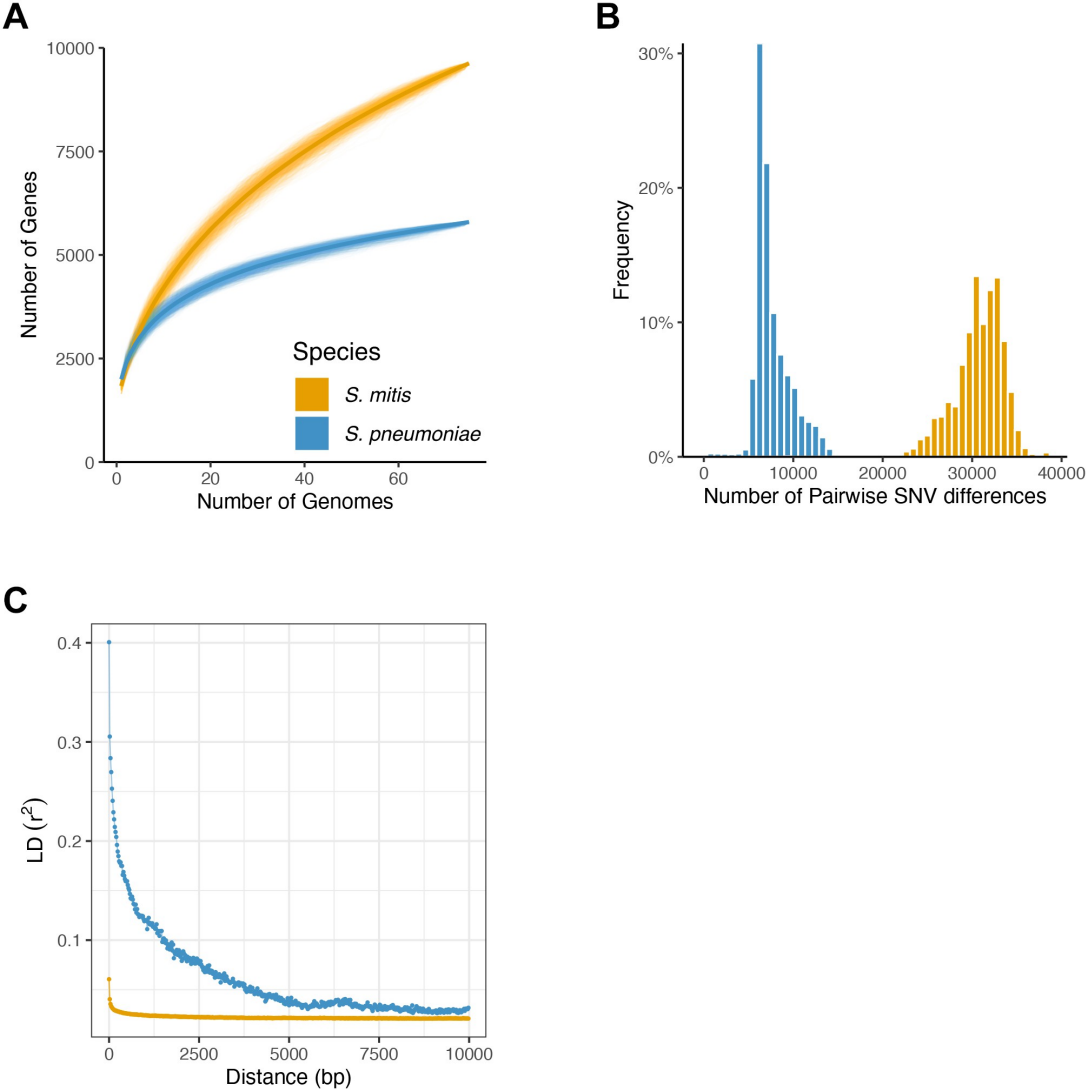
*S*. *mitis* and *S*. *pneumoniae* genetic variation. **A**. *S*. *mitis* and *S*. *pneumoniae* pangenomes according to power law fit. The number of genes is plotted as a function of the number of genomes. In *S*. *mitis*, the order of the 75 unrelated genomes was permuted 1000 times. In *S*. *pneumoniae*, the same procedure was applied to each of 1000 random samples of 75 unrelated genomes. Power law regression was fitted to the mean number of genes obtained across all permutations. Fitting the same model to the median gave similar results, albeit with lower goodness-of-fit to the *S*. *pneumoniae* data. The parameterisation that best fitted the data was: Y = *a*X^*b*^ + *c*. For *S*. *mitis*, *b* = 0.42; for *S*. *pneumoniae*, *b* = 0.12–0.32; mean = 0.2. **B.** Pairwise SNV differences distribution for *S*. *mitis* and *S*. *pneumoniae* after controlling for clonality (pairwise SNV differences threshold of 1000 for *S*. *mitis* and of 100 for *S*. *pneumoniae*). **C.** LD (*r*^*2*^) decay with distance. *S*. *mitis*, orange; *S*. *pneumoniae*, blue.

In *S*. *pneumoniae*, the presence of clonal relationships was detected both within and between hosts at a larger degree than in *S*. *mitis* (S1B and S1D Fig). The mean number of pairwise SNV differences between serotypes is 7,309 (IQR:6,310–7,748), similar to the mean number of pairwise SNV differences within serotypes (mean = 7,265, IQR: 5,348–10,481). Considering genomic variation in carriage *S*. *pneumoniae* organised in GPSCs, the mean number of pairwise SNV differences is greater between (7,393; IQR: 6,319–7,866) than within the defined clusters (1,880, IQR: 128–3,113). Analysing the within-the-host pairwise SNV differences distribution in the African sample, we observe that ~97% of the pairwise comparisons within GPSCs and within serotypes are smaller than 100; the remaining pairwise SNV comparisons are above the first quartile of between-host pairwise SNV differences. We then set a threshold of 100 pairwise SNV differences to define clonal relationships in *S*. *pneumoniae*, which maintains the serotype (n = 62), sequence type (n = 247) and GSPC clusters (n = 134) diversity across all geographic regions (Fig 1B). However, we also performed pangenome and core-genome diversity and population structure analyses with the more conservative thresholds of 1000 (as in *S*. *mitis*; 0.8% percentile of the pairwise SNV distribution), 2000 (1% percentile of pairwise SNV distribution) and 5600 (5% percentile of pairwise SNV distribution) pairwise SNV differences, to confirm that results and main conclusions are not affected by closer relatedness of few pairs of isolates (S2 and S3 Figs).

Considering the established cut-off for clonality for both species (1000 pairwise SNV differences for *S*. *mitis*; 100 pairwise SNV differences for *S*. *pneumoniae*), we observe higher within-host diversity for *S*. *mitis*: 64 out of 242 (26%) and 155 out of 236 (66%) within-host pairwise comparisons are defined as clonal relationships for *S*. *mitis* and *S*. *pneumoniae*, respectively. We can conclude that human hosts are generally colonised by multiple and divergent strains of *S*. *mitis*, whereas *S*. *pneumoniae* populations within the host are most often dominated by a single strain.

As we are interested in investigating global population genetic structure and long-term evolutionary dynamics of both species, we extracted the maximal datasets composed solely by unrelated strains (that is, datasets composed by isolates whose pairwise SNV differences are bigger than the cut-offs for clonality defined above). In total, 75 *S*. *mitis* isolates (25 African, 32 European, 18 Asian) from 45 hosts, and 353 *S*. *pneumoniae* isolates (78 African, 68 European, 207 Asian) from 339 hosts from across the three geographic regions were used in the analyses.

### *S*. *mitis* has considerably higher between-host diversity than carriage *S*. *pneumoniae*

Phylogenetic analyses of geographically restricted (mainly European) *S*. *mitis* and mainly invasive *S*. *pneumoniae* genomes have shown higher diversification of *S*. *mitis* than *S*. *pneumoniae* [15]. Here, we analyse if these patterns of genome variation for both species are observed in our extended dataset of exclusively carriage isolates from across geographically distant regions, which corresponds to analysing the scale of diversity in these species associated with host ancestry and with deeper evolutionary timescales.

We first evaluated the sets of genes available to both species for their evolutionary success. The pangenome–defined as the total set of genes observed across all sampled isolates—and mean individual genome sizes are similar across the three geographic regions for *S*. *mitis* and *S*. *pneumoniae* (S1 Table). To compare the pangenome and genome sizes between the two species, we have analysed 1000 random samples of unrelated *S*. *pneumoniae* with the same size as *S*. *mitis* (n = 75) and present the mean estimates. Individual genome size is smaller for *S*. *mitis*–on average 1832 genes (SD = 84) for *S*. *mitis* and 2005 genes (SD = 71) for *S*. *pneumoniae* (Mann-Whitney test, *P*-value< 2.20 x10^-16^). However, *S*. *mitis* gene repertoire is significantly larger than that of *S*. *pneumoniae*. In a total of 75 strains, the *S*. *mitis* pangenome was estimated to harbour 9626 genes, of which ~10% were found in all strains (core genes, 951 genes). At the same level of resolution, *S*. *pneumoniae* pangenome size is 6064 genes (mean across 1000 random samples; SD = 250), of which 18% comprise core genes (mean ± SD = 1092 ± 29). Furthermore, the impact of each additional genome on the size of the pan genome is greater for *S*. *mitis* than *S*. *pneumoniae* (Fig 1A). This is independent of any pairwise SNV cut-off considered to define clonality for *S*. *pneumoniae* (S2 Fig). Analysing permuted data with power law regression [54], we estimated that each *S*. *mitis* genome adds on average 2.8 times more genes to the pangenome than any *S*. *pneumoniae* genome (Fig 1A).

Based on the core genomes extracted for each species, nucleotide diversity (π) within species, estimated accounting for core genome length and sample size, was similar across the three geographic locations: for *S*. *mitis*, π values for the core genome are 0.034, 0.032 and 0.033 for the African, Asian and European bacterial populations of isolates, respectively; for *S*. *pneumoniae*, π values for the core genome are 0.008, 0.010 and 0.008 for the African, Asian and European bacterial populations of isolates, respectively. π values obtained for *S*. *pneumoniae* when considering higher thresholds of pairwise SNV differences were very similar (S2 Fig). Considering the species as a whole, π is higher in *S*. *mitis* (π = 0.034) than in *S*. *pneumoniae* (π = 0.010) (Fig 1B compares the distribution of pairwise SNV differences between species). *S*. *mitis* is therefore considered to have substantially higher genetic diversity than its close relative pneumococcus in terms of both pangenome content and sequence variation.

### Limited population differentiation in both *S*. *mitis* and carriage *S*. *pneumoniae*

**Fig 2:**
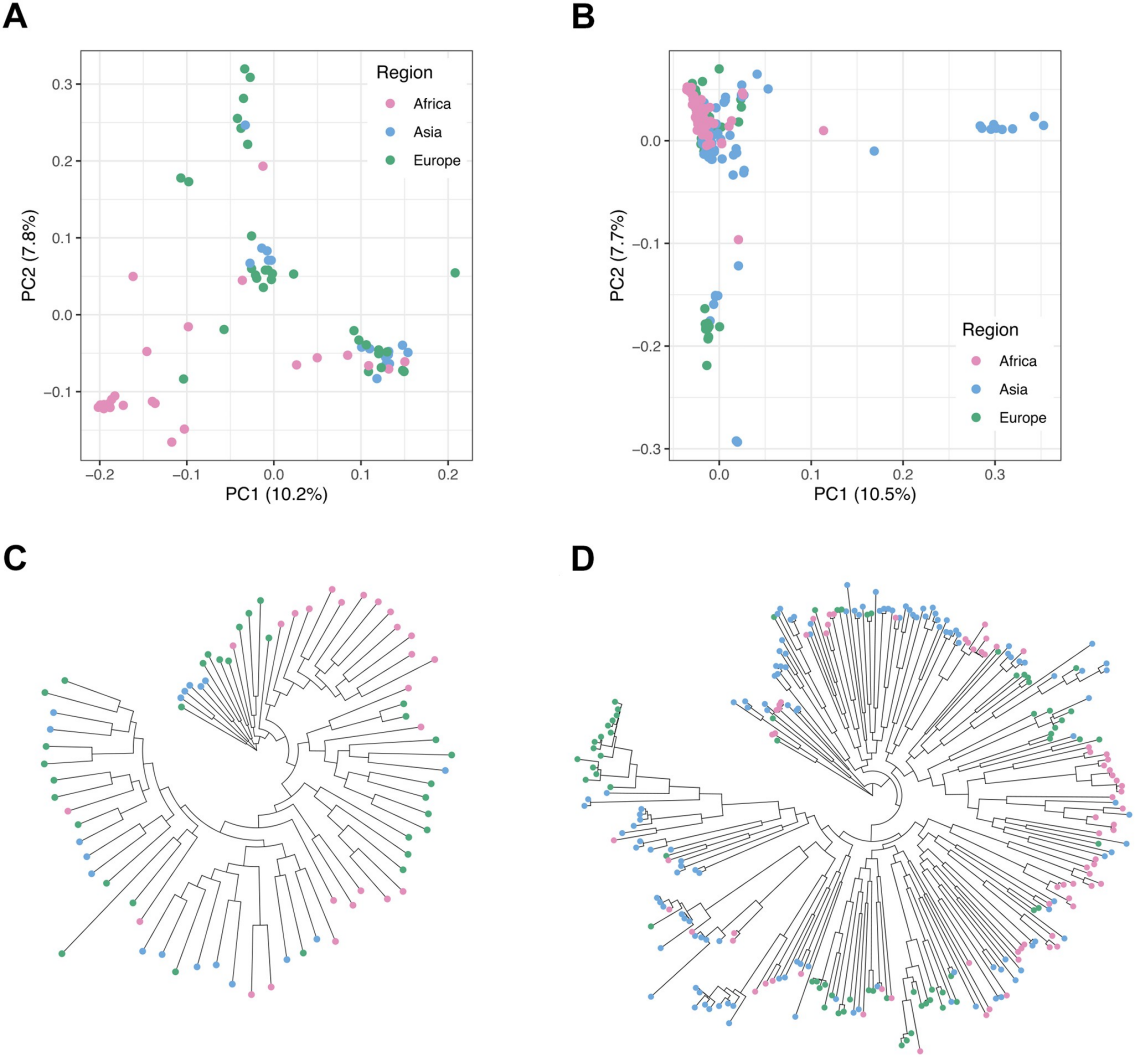
Population genetic structure in *S*. *mitis* and *S*. *pneumoniae*. **A.** plots of PC1 versus PC2 for *S*. *mitis*. **B.** Plots of PC1 versus PC2 for *S*. *pneumoniae*. **C.** Maximum likelihood unrooted phylogenetic tree for *S*. *mitis*. **D.** Maximum likelihood unrooted phylogenetic tree for *S*. *pneumoniae*. Analyses presented here for *S*. *pneumoniae* do not include outlying serotype NT isolates, whose higher genetic variation has been attributed to higher recombination rates for this clade [44]. Graphical representation of the analyses for the full *S*. *pneumoniae* sample are in S4 Fig. The two phylogenetic trees are in the same scale. Colour code corresponds to geographic region: Africa, pink Asia, blue; Europe, green.

Phylogenetic analysis and PCA show both species to have little genetic differentiation among geographic locations (Figs 2, S3 and S4). Concordant with these analyses, between bacterial population divergence values in *S*. *mitis* and *S*. *pneumoniae* among geographic regions are >3-fold lower (average Hudson’s F_ST_ = 0.043 in *S*. *mitis*; 0.039 in *S*. *pneumoniae*; S2 Table) than that in humans from the same geographic regions (average Hudson’s F_ST_ = 0.135; [55]). Analysis of molecular variance confirms that most of the genetic variation in *S*. *mitis* and *S*. *pneumoniae* is segregating within rather than between geographically distinct bacterial populations: the within-population component explains 95.3% and 96.3%, whereas the among-geographic-regions component explains 4.7% and 3.7% of the genetic variation, respectively for *S*. *mitis* and *S*. *pneumoniae*. One caveat in our analysis is that our *S*. *mitis* Asian sample was mainly collected in the UK and, therefore, might include one or two genomes that are closer to European genomes via horizontal transmission [12,56], lowering F_ST_ values between these two bacterial populations. However, these will not affect our overall observation of more within-population rather than between-population diversity.

We can conclude that, although the two species conform to contrasting population models of transmission and dispersal, both *S*. *mitis* and *S*. *pneumoniae* genomic variation is mostly randomly assorted according to geography and host ancestry. The lack of strong population differentiation in both species means that the evolutionary history of the isolates can be modelled by assuming that isolates form a single, non-structured population.

### Genetic diversity difference between *S*. *mitis* and *S*. *pneumoniae* is not due to mutation or recombination

The considerably higher level of *S*. *mitis* genetic diversity in comparison to *S*. *pneumoniae* can be attributed to deterministic (mutation and recombination) and population level processes (genetic drift and selection) which dictate the frequency of the allelic variation in the population. To evaluate the roles of mutation and recombination in driving bacterial variation in *S*. *mitis* and *S*. *pneumoniae*, we used the coalescence analytical framework implemented in mcorr [57], which is appropriate for species composed of highly divergent strains and for which the underlying phylogeny is difficult to estimate accurately. In addition, the estimation of evolutionary parameters in mcorr is based on synonymous variation only, which minimises the effect of selection.

Rates of recombination to mutation (*δ*/*μ*) were >1 for both *S*. *mitis* and *S*. *pneumoniae* (Table 1), showing recombination to have had a greater role in the species diversification than mutation in both species and replicating previous results for *S*. *pneumoniae* [39,58]. Mutational divergence (also known as effective mutation, $\theta_{\text{pool}}=\theta_{\text{pool}}=2\bar{T}\mu$, where *μ* is the mutation rate, and $\bar{T}$ is the mean pairwise coalescence time across all loci in the overall population and equals *N*_*e*_/2 in the mcorr coalescence model) is significantly higher than the sample’s diversity for both species, indicating that their population gene pool is highly diverse and that recombination within species can happen between highly divergent sequences (with divergences as high as 18% for *S*. *mitis* and 10% in *S*. *pneumoniae*; Table 1).

**Table 1:** mcorr estimates of recombination and mutation parameters for *S*. *mitis* and *S*. *pneumoniae*. n, sample size; n_genes_, number of genes analysed; *d*_sample_, diversity of the sample; *θ*_pool_, mutational divergence; and *ϕ*_pool_, recombinational divergence of the of the species’ gene pool; *δ*/*μ*, the relative rate of recombination to mutation; $\bar{f}$, the mean recombination fragment length; *c*, recombination coverage. A definition of these parameters is in Methods.

Species	n	n_genes_	*d*_sample_	*θ*_pool_	*ϕ*_pool_	*δ*/*μ*	$\bar{f}$ (bp)	*c*
*S*. *mitis*	75	950	0.046	0.18	0.82	4.61	2511	0.32
*S*. *pneumoniae*	353	972	0.012	0.10	0.14	1.41	1004	0.13

Although mutational divergence is similar between species (see also S3 Text), recombination divergence (or effective recombination, $\phi_{\text{pool}}=2\bar{T}\delta$, where *δ* is recombination rate) is considerably higher in *S*. *mitis* (Table 1). Recombination frequency in both species was also evaluated by analysing the decay of LD with physical distance (Fig 1C). Neutral (based on synonymous variation alone) LD *r*^*2*^ values for *S*. *pneumoniae* decay from a maximum of ~0.3 to low, considered non-significant, values—less than 0.1—within 1500bps; LD *r*^*2*^ maximum value is ~ 0.06 for *S*. *mitis* and halves within 150 bps. These values together with mcorr recombination parameters, indicate a history of frequent genetic exchange that is preventing the presence of strong population structure in both species. The low levels of neutral LD *r*^*2*^ for *S*. *mitis* are consistent with a quasi-sexual mode of evolution for this species [5].

Importantly, neutral LD *r*^*2*^ values in *S*. *mitis* are considerably lower and decay to minimal values faster than LD *r*^*2*^ in *S*. *pneumoniae* (Fig 1C). In non-structured populations such as the ones under study, neutral LD between alleles is maintained by a balance between genetic drift and recombination [59]. One explanation for the observed differences in long-range LD for both species is that recombination rates are higher in *S*. *mitis*. However, under this hypothesis, recombination to mutation rates in serotype lineages in *S*. *pneumoniae* would be expected to be consistently lower than in *S*. *mitis*, and this pattern is not observed [58]. Therefore, the most likely explanation for lower neutral LD in *S*. *mitis* is long-term evolution based on lower levels of genetic drift. This hypothesis is also consistent with the genomic diversity and population structure patterns observed in *S*. *mitis* (see Discussion) and is compatible with its quasi-sexual mode of evolution. In summary, the difference in genetic diversity between the two species is not due to mutation or recombination.

### Long-term evolutionary dynamics for *S*. *mitis* and carriage *S*. *pneumoniae*

**Fig 3:**
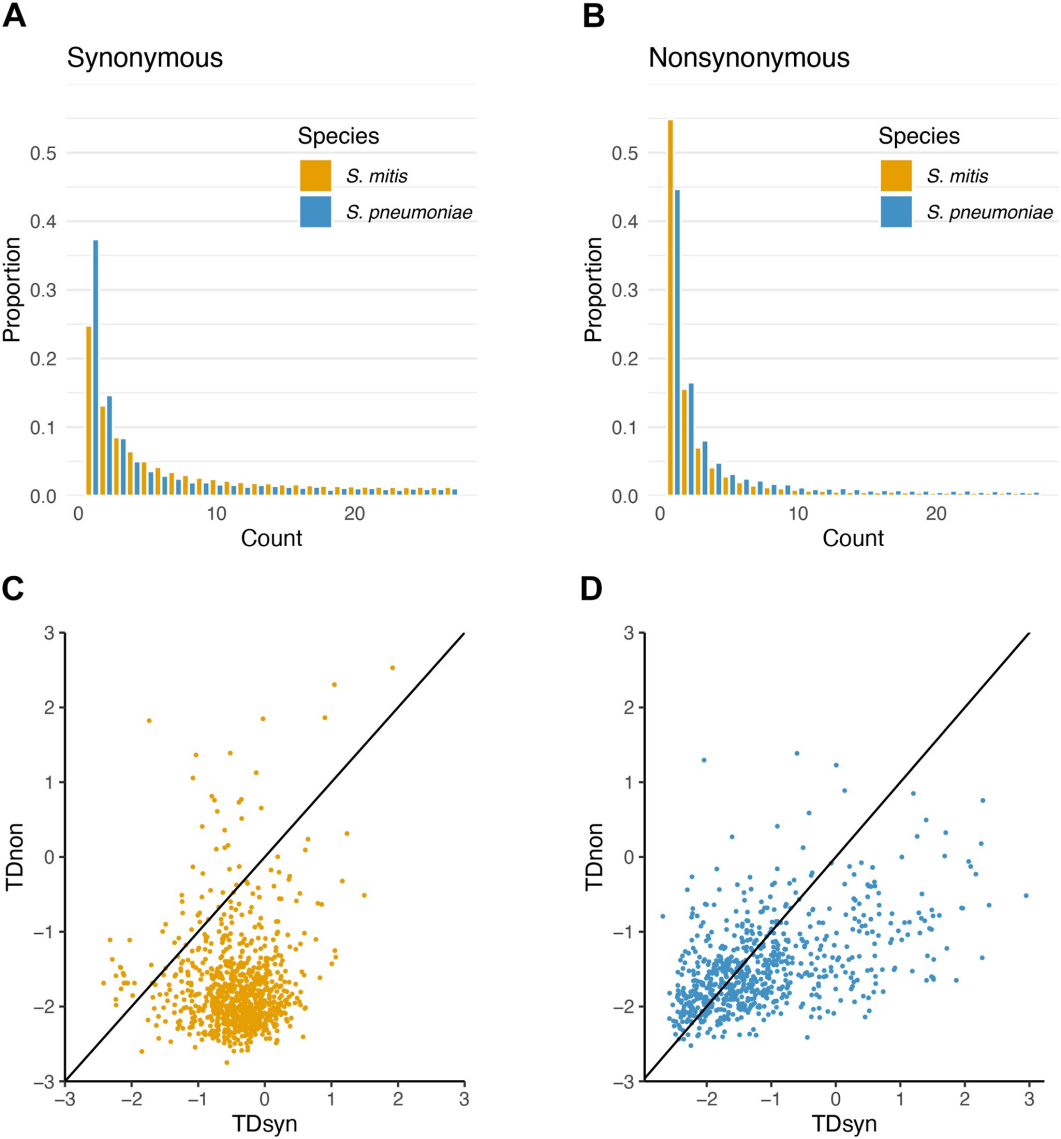
Evolutionary dynamics of *S*. *mitis* and *S*. *pneumoniae*. **A.** Folded site frequency spectrum for synonymous variation. **B.** Folded site frequency spectrum for nonsynonymous variation. To get a comparable result, the SFS was calculated based on the same sample size for both species. **C.** Tajima’s D for synonymous variation (TDsyn) vs Tajima’s D for nonsynonymous variation (TDnon) in *S*. *mitis*. Line corresponds to the diagonal (where x = y). **D.** Tajima’s D for synonymous variation (TDsyn) vs Tajima’s D for nonsynonymous variation (TDnon) in *S*. *pneumoniae*. Line corresponds to the diagonal (where x = y). *S*. *mitis*, orange; *S*. *pneumoniae*, blue.

We computed the minor-allele frequency distribution of variant sites, called the folded site-frequency spectrum (_f_SFS; Fig 3A and 3B), independently for synonymous and nonsynonymous variation, each to evaluate the role of genetic drift (derived from demographic history of the species) and selection occurring during each species’ evolutionary history, respectively [60]. Notably, because we inferred high effective recombination for both species (Table 1), we assume that selection only affects a reduced number of closely linked neutral sites around the selected variant.

*S*. *pneumoniae* shows an excess of rare synonymous (<2%) variants compared to *S*. *mitis* (chi-square (1df), p-value < 2.20 x10^-16^) (Fig 3A). In contrast, a higher number of intermediate frequency synonymous variants (>5%) is observed in *S*. *mitis*. Conversely, the overall *S*. *mitis* nonsynonymous _f_SFS is skewed towards rare variation in comparison to *S*. *pneumoniae* (chi-square (1 df), p-value < 2.20 x10^-16^; Fig 3B). The distributions of synonymous variation suggest contrasting demographic models of non-growth versus population growth in *S*. *mitis* versus *S*. *pneumoniae* and the distribution of non-synonymous variation, potentially, different types and magnitudes of selection.

To test for these hypotheses, both species’ _f_SFS were summarised by calculating Tajima’s D (TD) per core genome and per core gene, for synonymous (TDsyn) and nonsynonymous (TDnon) variants (Fig 3C and 3D). Overall core genome TDs were compared with simulated TDs obtained under a standard population equilibrium and selective neutral model (Methods). In *S*. *mitis*, TDnon is consistently smaller than TDsyn (Fig 3C). The overall TDsyn is -0.36 (p-value = 0.380), whereas TDnon is -1.91 (p-value = 0.003). This confirms that the evolutionary model most consistent with *S*. *mitis* genomic variation is one of a stationary population in equilibrium (at least over many generations) and natural selection acting at non-synonymous sites. In contrast, there is a linear tendency for larger values of TDnon to be associated with larger values of TDsyn in *S*. *pneumoniae*, and both overall metrics are significantly smaller than zero (overall TDnon = -1.90, p-value = 0.001; overall TDsyn = -1.40, p-value = 0.039). This confirms that the _f_SFS of *S*. *pneumoniae* is more consistent with a model of population growth influencing the scale of genetic diversity observed in *S*. *pneumoniae* and natural selection acting at nonsynonymous sites.

We further investigated the demographic history of *S*. *pneumoniae* and *S*. *mitis* using an approximated Bayesian computation (ABC) framework. For each species, we simulated 30,000 250-Kb windows for *S*. *mitis* and 75,000 250-Kb windows for *S*. *pneumoniae* with sample size and number of synonymous (neutral) SNVs matching the ones estimated for overlapping sliding windows of the same size across the sampled genomes (overlapping by 200 Kb) and whose SFS is derived from an analytical approach [61,62] that incorporates the Kingman coalescence model with exponential growth characterized by a specified growth rate *g* (including no growth (*g* = 0), that is constant population sized). We then compared the genetic diversity obtained from the simulations with the observed genetic diversity via a Random Forest ABC classifier to get the posterior growth rate parameter distributions (see Methods and S1 Text). This approach does not model recombination (we assumed each SNV to be independent due the absence of significant LD in both species; Fig 1C); however, running ABC on genetic diversity of genomic windows simulated under an exponential growth model and incorporating the recombination rate estimated for *S*. *pneumoniae* gave similar results (S2 Text).

Our approach shows support for significant population growth in *S*. *pneumoniae* (median growth rate = 2.7; 95% confidence interval, 95% CI = [1.4–9.6], in coalescent time units of *N*_*e*_; Table A in S1 Text). In contrast, the model that best fits *S*. *mitis* genetic diversity includes a very small, non-significant, (median) growth rate of 0.25 (95% CI = [0.2–0.4], Table B in S1 Text), compatible with an evolutionary model with very low growth or with one of stationary population for at least many generations.

A sliding window analysis (with sizes of 250Kb, overlapping by 50Kb, or 100Kb, overlapping by 10Kb as for our ABC analysis; S1 and S2 Texts) of TDnon and TDsyn across *S*. *mitis* and *S*. *pneumoniae* genomes showed that TDnon is consistently more negative than TDsyn (e.g. for 100Kb windows, TDnon-TDsyn ranges between [-0.84, -0.16] in *S*. *pneumoniae* and [-1.84, -1.26] in *S*. *mitis*). This overall trend is evidence for widespread purifying selection in both species leading to rarer non-synonymous alleles (in contrast with synonymous alleles), that reduce the non-synonymous nucleotide diversity in comparison to the number of non-synonymous segregating sites, in this way, leading to more negative TDnon. Of note, we do not expect directional selection to contribute meaningfully to this signature in these highly recombining species due to the absence of genetic hitchhiking.

To further investigate the role of natural selection in *S*. *mitis* and *S*. *pneumoniae*, we analysed two summary statistics of genetic diversity for all genes and compared them with null distributions obtained from sampling randomly non-synonymous and synonymous variants from across the two species’ genomes (see Methods). We first analysed the ratio of the number of non-synonymous variants to the number of synonymous variants [46] and observed a significant fraction of genes with an excess (p-value<0.05; corresponding to the action of diversifying selection) and deficiency (p-value>0.95; corresponding to the action of purifying selection) of non-synonymous variation in both species (binomial test p-value<2.2e-16; S5 Fig). We also evaluated the ratio of nucleotide diversity for non-synonymous to nucleotide diversity for synonymous variation (*π*_*N*_/*π*_*S*_) and, in this case, both species’ gene distributions show smaller non-synonymous genetic diversity in comparison to the null distribution (S6 Fig). Altogether, we can conclude that the signature of diversifying selection influencing the genetic diversity in both species is not leading to an excess of intermediate-frequency non-synonymous variants.

### Long-term evolution of effective population sizes

Neutral genetic diversity within populations is determined by the population effective size and the mutation rate, a notion that is captured in the population parameter *θ*. As we confirmed experimentally that the mutation rate and growth cycle are comparable between the two species (S3 Text), differences in *θ* between *S*. *mitis* and *S*. *pneumoniae* will be due to their *N*_*e*_. We have then inferred the coalescent *N*_*e*_ using the observed number of segregating sites for each species (which ≈ *θE*(*L*_*n*_), where *E*(*L*_*n*_) is the expected length [in coalescent time units of *N*_*e*_] of the sample’s genealogy), and incorporating estimated growth rates to control for the demographic history in both species (see Methods). Considering the mutation rate from [35], this indicated that *S*. *mitis*’s contemporary *N*_*e*_ to 2.8x higher than *S*. *pneumoniae*’s *N*_*e*_: *S*. *mitis N*_*e*_ = 274,288 (IQR = [268,555–279,736]); *S*. *pneumoniae N*_*e*_ = 96,597 ([93,851–99,167] calculated based on the median, and minimum and maximum growth rates—S2 Text). We conclude that the ancestral population of *S*. *pneumoniae* prior to expansion was significantly smaller than the concurrent *S*. *mitis* population and that *S*. *pneumoniae*’s population expansion was not sufficient to recover similar contemporary *N*_*e*_ or genetic diversity levels to *S*. *mitis*.

## Discussion

Here, we have studied the genomic diversity, population differentiation and evolution of two phylogenetically related human-associated bacterial species, whose modes of transmission, dispersal, and interaction with host lead to different expectations in the magnitude of genomic variation (higher for *S*. *mitis*) and of population differentiation/structure (more significant for *S*. *mitis*). Indeed, we have determined higher historical *N*_*e*_ in *S*. *mitis* that has led to more substantial levels of genomic diversity in this species compared to *S*. *pneumoniae*, even though contemporary *N*_*e*_ between species is more comparable. However, both species present limited geographic differentiation across host populations contrary to our expectations based on modes of transmission. We now discuss how different patterns of natural colonization, transmission, dispersal, interactions with the host, and demographic histories have led to these populational patterns.

Contrary to *S*. *pneumoniae* (our data; [47]) and common species in the gut microbiome [5,63], where a dominant strain is retained over prolonged periods of the host life, our results show that *S*. *mi*tis exhibits multiple colonization. According to [13] diversifying lineages may coexist for long stretches of time, generating within-host variation that will be able to respond to strong selection pressures such as changes in diet or antibiotic intake. The presence of multiple highly divergent lineages within the same host makes *S*. *mitis* less amenable to be studied using metagenomic samples; here we developed a culture system that allows to obtain isolates for within-the host studies more effectively. Our data confirms that additional *S*. *mitis* lineages are acquired within households but can also be acquired horizontally within contained social networks, as recently described for the oral microbiome more generally [12,56]. Multiple colonization and some degree of horizontal transmission leads to effective recombination between strains (S1 Text), contrary to what was proposed [14].

We replicate the previously observed higher levels of genome variation in *S*. *mitis* than in *S*. *pneumoniae* [14] and extend these observations to the pangenome level. We determined that *S*. *mitis* variation is consistent with the most common evolutionary model for human commensals of population in mutation-drift equilibrium, with some degree of purifying selection [4,5]. The higher *N*_*e*_ calculated for this species leads to a highly diverse populational gene pool (S1 Text) within which horizontal gene transfer (HGT) can occur. In addition, our results go in agreement with recent evidence indicating that *S*. *mitis* pangenomes are shaped by cross-species HGT [14,64]. Although the *S*. *mitis* pangenome diversity can be explained by neutral evolution [65], future work should indicate if common accessory genes confer adaptive advantage within and across *S*. *mitis* populations [66].

Modelling of *S*. *pneumoniae* genomic diversity agrees with a population expansion as a demographic model for *S*. *pneumoniae*. As *S*. *mitis* and *S*. *pneumoniae* have the same host, the distinct demographic history between both species must be linked with behavioural differences. As [15] proposes, frequent horizontal transmission makes *S*. *pneumoniae* more dependent on having enough hosts for successful dispersal and, consequently, population growth in S. *pneumoniae* is most likely intimately linked with the increase in human population density [67]. In addition, we argue that the horizontal transmission features of continuous founding bottlenecks and recovery are analogous to range expansion processes, and these properties can lead to the same genomic patterns as an (instantaneous) population expansion if the number of ‘migrants’ or bacterial cells between hosts is large (*Nm*>>1, where *N* is the within-host bacterial population effective size; [68]). Within-host deep-sequencing showed transmission bottlenecks sizes between donor and recipient to be higher than one [47], the within-host diversity of one colonization event (defined from cultured isolates) is consistent with *N*_*e*_ ranging from 1 to 72 bacterial cells [46], and mice colonization experiments allowed to estimate within-host *N*_*e*_*s* of ~100 [69]. In fact, a multiple merger coalescent genealogy was previously inferred for *S*. *pneumoniae* [62], which is concordant with range expansion [70]. It would be interesting to understand how between-host transmission affects *N*_*e*_ over a great number of generations. Another aspect of *S*. *pneumoniae*’s genetic diversity is that it is also due to purifying selection albeit to a lower degree than *S*. *mitis* (S5 Fig).

An important question that has been debated recently is the amount of commensal strain variation within and among different geographic regions or ethnic groups [17,18]. Here we show that two important components of the oro-nasopharyngeal microbiome in humans show little differentiation across human populations. In an island model under neutral equilibrium, F_ST_ is equal to 1/(1+2*N*_*e*_*m*) in haploids where *m* is the migration rate [50]. Studies on the transmissibility of the oral genome showed that strain dispersal across human populations is minimal but not null [12]. Therefore, for *S*. *mitis* the large *N*_*e*_ leads to values of *N*_*e*_*m* >>1, even if *m* is very small, reducing the differentiation among human populations. In contrast, there are reports of pathogenic outbreaks extending for long geographic distance within Europe and across the world for *S*. *pneumoniae* [35,71], and we detected pairs of highly related strains between continents (within our dataset 350 pairs of strains involving 125 strains from different geographic regions had <2000 SNV pairwise differences [<1%percentile]), demonstrating meaningful migration rates. Therefore, although *N*_*e*_ is smaller in *S*. *pneumoniae*, it may have been and currently is sufficiently high enough that together with higher *m* make *N*_*e*_*m* >>1. Estimates of *N*_*e*_, HGT, between-host transmission and of dispersal are known to vary among commensal species, but there is evidence for a great fraction of the human microbiome that they are high enough to lead to the same patterns as in *S*. *mitis* and *S*. *pneumoniae* [4,12,56,72,73]. In conclusion, our results support the view that more diversity within than among human populations and little population differentiation must be common features of the human microbiome due to large *N*_*e*_.

## Material and methods

### Ethics statement

African *S*. *mitis* samples were collected by MRC The Gambia Unit, under ethical approval granted by The Gambia Government/Medical Research Council Joint Ethics Committee.

European and East Asian samples were collected under ethics approval granted by Ethics Committee for Medicine and Biological Sciences at the University of Leicester (protocol 14610). All samples were collected from Live Participants in Leicester, UK, and all participants gave their written consent.

### Bacterial isolate collection

Buccal swabs are part of an extended dataset collected from across The Gambia from family trios consisting of mother, child (3–10 years old) and a baby (less than 2 years old). Suspected streptococcal species (based on plate morphology and presence of alpha-haemolysis) were analysed via MALDI-TOF mass spectrometry, and putative Mitis group isolates had their genomes sequenced (see below). Mitis group species identification was done analysing MLSA variation as in [74]. The final dataset consisted of 32 isolates from one baby/two siblings’ trio, two sibling/baby duos, two mother/child duos, and six unrelated individuals.

European samples were collected from the cheeks and tongue individuals natural and living in the UK for the past 2–5 years. East Asian samples were collected from five Chinese individuals that had lived in the UK for less than six months, maintained a Chinese diet and had either no romantic partner or a Chinese partner who met the same criteria. We complemented these datasets with publicly available European and East Asian whole genomes from NCBI-SRA (https://www.ncbi.nlm.nih.gov/sra; S3 Table), which we confirmed via phylogenetic and F_ST_ analyses that they did not differ significantly from our two sample sets.

*S*. *pneumoniae* carriage samples matching the broad geographic origin of the *S*. *mitis* dataset (Europe: UK, East Asia: Thailand and Africa: The Gambia) were collated from the literature [42,44,46]. Serotypes and sequence types are disclosed in respective publications and genomic sequences are available on the European Nucleotide Archive under study accessions: PRJEB2357 (Asian dataset), PRJEB2417 (European dataset), and PRJEB3084 (African dataset). We only analysed isolates from non-vaccinated individuals. For the East Asian dataset, we considered the subset of isolates collected in the first year of a 3-year sampling period, to obtain a random sample of a size comparable to the other studied geographic regions. This subsample might include more than one isolate per individual, but because we did not have access to that information, we did not consider it for within-host analyses. For the African and European datasets, we considered only the within-host carriage isolates that were collected pre-vaccination.

### *S*. *mitis* isolation and growth

The European and East Asian samples were cultured on Mitis Salivarius Agar (5% Sucrose (Fisher Scientific), 1.5% Peptone (Oxoid), 1.5% agar (BioGene), 0.5% Tryptone (Oxoid), 0.4% di-potassium hydrogen orthophosphate (Fisons), 0.1% Glucose (Fisons), 7.5E-3% Trypan Blue (Sigma), 8E-5% Crystal Violet (Sigma)) [75]. Putative *S*. *mitis* colonies were identified based on flat, “rough” morphology and a blue colouring in the centre of the colony following 18 hours growth at 37°C, 5% CO2, and on the sequencing of the house keeping gene *glucose-6-dehydrogenase* (*gdh*) for unambiguous differentiation between Mitis group species [13].

This isolation technique had a significantly better yield than that used for African samples: 95% of isolates predicted as *S*. *mitis* based on *gdh* phylogeny clustering were confirmed as *S*. *mitis* based on whole genome data (European and East Asian isolates) compared to 37% of isolates where species was predicted with MALDI-TOF mass spectrometry (African isolates).

### DNA extraction, sequencing and *de novo* sequence assembly

DNA extraction was performed using Wizard Genomic DNA purification kit (Promega) and manufacturer’s instructions for Gram-positive bacteria. Samples were sequenced on an Illumina HiSeq 4000 at the Oxford Genomics Centre (UK), to a mean coverage of 83.4x for African isolates and 300x for East Asian and European isolates.

Raw FASTQ reads were quality normalised with Trimmomatic 2.0 [76]. Quality was confirmed via FastQC 0.11.5 [77]. Trimmed, paired reads were assembled to contig level using SPAdes 3.12 [78]. Assembly was improved to scaffold level using the “assembly improvement” pipeline from Sanger Pathogens [79]. Assembly quality was assessed via QUAST 4.3 [80]. The mean N50 across isolates was 239.6Kb (42.3Kb-931Kb) and the GC% content (39.2%-40.6%, mean = 40.1%) was mirrored across isolates. Accession numbers for this dataset are in S3 Table.

### Serotyping and determination of Global Pneumococcal Sequence Clusters

Capsular serotyping and determination of Global Pneumococcal Sequence Clusters (GPSCs) of shared descent for *S*. *pneumoniae* [52,53] were performed by uploading newly assembled genomes in the web tool PathogenWatch (https://pathogen.watch/). Two genomes from the Asian *S*. *pneumoniae* dataset were classified as *S*. *pseudopneumoniae* and were therefore removed from the dataset.

### Core and accessory genome extraction and pangenome analysis

Scaffolds were annotated using Prokka 1.14.6 with default settings [81] and the full set of non-redundant genes was extracted with Roary 3.13 [82]. Alignment was made invoking MAFFT [83] and considering a sequence identity of 85%. The percentage sequence identity was empirically determined, by considering the percentage that minimised the change in the number of genes per change in the parameter. Core genomes were formed as a concatenation of genes identified to be present in 100% of input sequences. The total core genome sizes obtained were 900,605 bp for *S*. *mitis* and 816,449 bp for *S*. *pneumoniae*.

To evaluate differences of pangenome size between *S*. *mitis* and *S*. *pneumoniae*, we performed an iterative procedure in which we permuted the input order of the bacterial genomes and assessed the rate of new genes identified per addition of each genome. For *S*. *mitis*, the input order of the (unrelated) genomes was permuted 1000 times. For *S*. *pneumoniae*, because final sample size of (unrelated) isolates was significantly larger than for *S*. *mitis*, we employed the same procedure to each of 1000 random samples of unrelated genomes of the same size as the one for *S*. *mitis*. Obtained curves were fitted applying power law regression to the mean number of number of genes obtained across permutations [54].

### Phylogenetic analysis

Phylogenetic trees were built from FASTA alignments with FastTree 2.1 [84], using the generalised time-reversible model of nucleotide evolution and re-scaled based on likelihoods reported under the discrete gamma model with 20 rate categories. This is a standard approximation for accounting for variable evolutionary rates across sites and uncertainty in these rates [85]. Trees were visualised using iTOL [86].

### Variant and gene mapping and annotation

Trimmed fastq files were mapped to type strains *S*. *mitis* NCTC 12261 (NCBI accession: NZ_CP028414.1) and *S*. *pneumoniae* R6 (NCBI accession: NC_003098.1) using BWA mem, and variants called using SAMtools 1.3.2 mpileup [87,88]. In order to be called a core variant, a site read depth of >10% of the mean genome-wide read depth, as well as minimum sequencing and mapping quality scores of 30, were implemented.

Synonymous and nonsynonymous variants were called in comparison to the type strains using snpEff [89]. To generate genetic diversity estimates per gene (see below), we considered the gene coordinates recorded in the genome assemblies of type strains.

### Genetic diversity, population structure, and folded site frequency spectrum analyses

These analyses used biallelic variant sites only. Pairwise SNV differences were calculated with the software SNP-dists 0.6 [90]. Pairwise F_ST_ between populations and nucleotide diversity (π) of core genomes were calculated with the Python package scikit-allel [91]. We calculated Hudson’s F_ST_ estimator which is less influenced by differences in sample size and SNV ascertainment scheme [55]. Pairwise F_ST_’s are based on the set of SNVS segregating in both populations; however, considering the set of SNVs segregating in either population gave similar F_ST_ values. Analysis of Molecular Variance (AMOVA) based on the pairwise differences between sequences was performed in ARLEQUIN ver3.5.2.2 [92].

Principal component analyses (PCA) of population structure were conducted in PLINK v1.9 [93]. PLINK V1.9 was also used to determine the Minor allele frequency (MAF) of variant sites, which was used to generate the _f_SFS for synonymous and nonsynonymous sites.

### Estimation of mutation and recombination parameters

Recombination and mutation populational parameters were estimated using mcorr [57]. Aligned-sequenced gene fasta files resulting from our Prokka/Roary/MAFFT pipeline were converted to XMFA files [94], which were used as input files in mcorr. Only sequences with <2% gaps of the total alignment length were used in the analysis. Mcorr computes the correlation profile, P(l), in each species by averaging over the correlation profiles of synonymous substitutions for all gene sequence pairs in the sample, and then averaging over all genes. When homologous recombination is present, the probability of observing a correlated substitution (P(l)) decreases with distance between any two loci (l), at a rate that is proportional to the recombination rate. Otherwise, the function is constant. By fitting P(l) to an analytical form inferred based on a coalescence model with recombination, mcorr estimates parameters that characterize the more diverse species’ gene pool with which the sampled genomes have recombined: the mutational divergence ($\theta_{\text{pool}}=2\bar{T}\mu$, where *μ* is the mutation rate), the recombinational divergence ($\phi_{\text{pool}}=2\bar{T}\delta$, where *δ* is recombination rate), the relative rate of recombination to mutation (*θ*_pool_/*ϕ*_pool_ = *δ*/*μ*), and the mean recombination fragment length $\left(\bar{f}\right)$, where $\bar{T}$ is the mean pairwise coalescence time across all loci in the overall population $\left(\bar{T}=Ne/2\right)$ in the considered coalescence model). It also calculates the average fraction of the sampled genomes that were brought in by recombination (recombination coverage, *c*). Mcorr was implemented with default settings and 1000 bootstraps.

We also computed the decay of Linkage disequilibrium (LD) with physical distance. The LD statistic *r*^*2*^ between all pairs of synonymous SNVs with minor allele frequency bigger than 0.01 was calculated up to a distance of 10 Kb (—*ld-window-k 10*) with PLINK 1.9, using the command flags—*ld-window 5000* to allow computation of *r*^*2*^ between all SNVs, and—*ld-window-r2 0* to obtain the tabling of all r2 values (>0).

### Per gene analyses

Nucleotide diversity (π), Watterson’s θ, and Tajima’s D (TD) per gene and for synonymous and non-synonymous substitutions independently using scikit-allel. We tested if the TD statistic deviated from a population equilibrium and selective neutral model by generating 10,000 random samples under this model and with the same diversity as the one observed for both species in ARLEQUIN ver3.5.2.2 [92]; p-values of the D statistic correspond to the proportion of random D statistics less or equal to the observation. If this fraction is <0.05, we concluded that the observed TD is significantly different from zero and not evolving according to the standard neutral model. We then used observed TDsyn statistics to determine the action of specific demographic processes (TDsyn<0 is expected under population growth, and TDsyn>0 under population contraction), and observed TDnon statistics to determine the action of natural selection (TDnon< 0 is expected under purifying and positive selection and TDnon>0 is expected under balancing selection) on the species’ genetic variation.

To build null (selectively neutral) distributions of genetic variation in *S*. *mitis* and *S*. *pneumoniae* populations and test for the presence of natural selection, we took advantage of the fact that bacterial genomes are gene heavy and extracted the synonymous and non-synonymous variants from random sites across *S*. *mitis* and *S*. *pneumoniae* genomes to build randomly sampled windows. We only retained windows that have a minimum of one non-synonymous variant and one synonymous variant. We have then calculated two summary statistics of genetic diversity: the ratio of the number of non-synonymous variants to the number of synonymous variants and the ratio of the nucleotide diversity for non-synonymous variation to the nucleotide diversity for synonymous variation (*π*_*N*_/*π*_*S*_). These were then compared with observed ratios at genes with more than one non-synonymous variant and one synonymous variant as well.

Specifically in the case of the ratio of the number of non-synonymous variants to the number of synonymous variants, for a gene of length *n*, we randomly picked *n* sites from across the genome and counted the number of non-synonymous and synonymous variants. This process was repeated 1,000 times for each gene in each species. We then calculated the fraction of simulated ratios that were bigger than the observed ratio, giving us a p-value for an excess of non-synonymous variants for each gene. We then tested if the fraction of genes with p-value<0.05 was more significant than expected under the by performing a binomial test, assuming a null distribution of uniform p-values.

In the case of *π*_*N*_/*π*_*S*_, we calculated *π*_*N*_ and *π*_*S*_ independently in 120,000 as follows. We first randomly sampled 1Kb windows (~mean size of *S*. *mitis* and *S*. *pneumoniae* genes), recorded the observed counts of non-synonymous and synonymous variants. We then extracted as many as non-synonymous and synonymous variants as in each sampled window randomly from across the genome and calculated *π*_*N*_/*π*_*S*_ with for these random SNV sets. We then compared this distribution with the observed distribution of *π*_*N*_/*π*_*S*_ for all genes.

### ABC computation

As high (*S*. *pneumoniae*) to very high recombination rates (*S*. *mitis*) lead to noticeably strong LD decay (Fig 1C), we assumed a simplified model where all synonymous SNVs are in linkage equilibrium and, therefore, appear on independent coalescence trees (this is, essentially, the Poisson Random Field approach from [95]). On a specific coalescent tree, a SNV has frequency *i* (that is, it is present in *i* sequences in the sample) with probability $B_{i}/\sum_{j=2}^{n}B_{j}$, where *B*_*i*_ is the summed length of all genealogical branches that are connected to exactly *i* sampled lineages. Thus, if we observe many SNVs (as in a large window), the proportion of SNVs that show a specific allele frequency is approximately given by ${E(B}_{i}/\sum_{j=2}^{n}B_{j})\approx {E(B}_{i})/\sum_{j=2}^{n}E(B_{j})$ (law of large numbers). Here, we used the analytical approach from [61] as implemented in [62] that uses a Kingman coalescence model with exponential growth of specified growth rate *g* to compute *E*(*B*_*i*_) and, in this way, generate a theoretical SFS under this model. Growth rates were drawn from a uniform prior distribution on the discrete set of *g* ∈[0,25] with step 0.1 (in coalescence units of *N*_*e*_ generations). We then obtained 30,000 simulated 250Kb windows for *S*. *mitis* and 75,000 simulated 250Kb windows for *S*. *pneumoniae* with the number of SNVs drawn randomly from the observed number of (synonymous) SNVs in sliding windows of the same size (with an offset of 50 Kb between adjacent windows) across the *S*. *mitis* and *S*. *pneumoniae* core genomes, and with a SFS derived from the described coalescent model with a specified growth rate *g*. We used a windowed approach to assess whether the genomic signal is stable across the genome. For *S*. *pneumoniae*, we restricted the analysis to the windows mapping to the first 950 Kb of the reference genome, where read coverage is consistently high (>10% of mean depth of coverage) among all isolates (encompass a continuous genomic region of the core genome), and where the number of observed segregating sites in consistent across windows (S1 Text).

For every simulated and observed window, a set of summary statistics of genetic diversity was recorded: the 5% percentiles of the minor allele frequencies; the 10% percentiles of pairwise Hamming distances between sequences, scaled by their maximum within the sample (simulated or observed); and Tajima’s D. Calculations on observed data were based on synonymous (neutral) variation.

To estimate posterior distributions and median point estimates of the exponential growth rate, we used the Random Forest ABC model selection procedure (ABC-RF) from the *abcrf* R-package [96]. To evaluate the accuracy of the model, we report the absolute error for simulations with growth rate *g* = 0 (averaged over all true simulations with *g* = 0) and the normalized mean absolute error NMAE, which averages |estimated value–true value|/true value across all simulations, for all other true growth rates combined (S1 Text).

To assess whether the model fits the observed genetic diversity, we performed graphical posterior predictive checks by calculating the Tajima’s D of simulated 1,500 250Kb-windows obtained using as parameters the 25% percentile, the median, and the 75% percentile of the estimated growth rates, and comparing these values with the observed Tajima’s D of the corresponding windows from which the growth rates were extracted from (S1 Text).

Scripts for running this analysis are available on GitHub at https://github.com/fabfreund/strepto_demography.git.

### *N*_*e*_ calculation

*N*_*e*_ and magnitude difference in *N*_*e*_ between species was calculated using coalescence theory and the observed number of segregating sites, *S*_*n*_. Ignoring the effect of selection, a sample of size n from a haploid species is expected to have E(*S*_*n*_) ≈ *S*_*n*_ = N_e_*mu*E(L_n_), where E(L_n_) is the expected length (in coalescent time units) of the genealogy of the sample [97], and *mu* is the per-genome, per-generation mutation rate (*mu* = *μgl*, where *μ* is the pneumococcal mutation rate of 1.57 × 10^−6^ site^−1^year^−1^ [35], *g* is the generation time of 14/cell divisions /year [46], and *l is* the core genome size for *S*. *mitis* and *S*. *pneumoniae*). We have then inferred *N*_*e*_ considering a population expansion model for both species, where E(L_n_) was calculated using the recursion from [98], as implemented in [99]. Scripts for running this analysis are available on GitHub at https://github.com/fabfreund/strepto_demography.git.

## Supporting information

S1 Text Accuracy and model fit of the ABC approach used to evaluate *S*. *mitis* and *S*. *pneumoniae* demographic histories. **Fig (i). Posterior predictive checks of the ABC approach implemented to investigate *S*. *mitis* demographic history**. The density plots show, from left to right, the distribution of Tajima’s D obtained from simulating 1,500 250Kb-windows (blue) using as parameters the 25% percentile, the median, and the 75% percentile of estimated growth rates from across windows, and the corresponding to the observed Tajima’s D estimated across the 34 windows considered (Table A in S1 Text). **Fig (ii). Posterior predictive checks of the ABC approach implemented to investigate *S*. *pneumoniae* demographic history**. The density plots show, from left to right, the distribution of Tajima’s D obtained from simulating 1,500 250Kb-windows (blue) using as parameters the 25% percentile, the median, and the 75% percentile of estimated growth rates from across windows, and the corresponding to the observed Tajima’s D estimated across the 15 windows considered (Table B in S1 Text). **Table A. Observed genetic diversity indices (S and Tajima’s D) and posterior estimates of growth rate obtained in the ABC-RF approach implemented to investigate *S*. *mitis* demographic history.** Presented are the median, 2.5% and 97.5% percentiles of growth rates obtained from 30,000 simulations. S, number of segregating sites. **Table B**. **Observed genetic diversity indices (S and Tajima’s D) and posterior estimates of growth rate obtained in the ABC-RF approach implemented to investigate *S*. *pneumoniae* demographic history.** Presented are the median, 2.5% and 97.5% percentiles of growth rates obtained from 30,000 simulations. S, # of segregating sites.
(PDF)

S2 Text Assessing the influence of recombination in the estimation of growth rates for *S*. *pneumoniae* via the ABC approach in FastSimBac. **Fig (i). Comparison between the distribution of observed number of segregating sites (pink; across 83 *S*. *pneumoniae* core genome windows) and the distribution of simulated number of segregating sites used in the ABC approach implemented to investigate *S*. *pneumoniae* demographic history.** The distribution of simulated S values follows the observed distribution closely. S, number of segregating sites. **Table A**. **Observed genetic diversity indices (S and Tajima’s D) and posterior estimates of growth rate obtained in the ABC-RF approach that considers all independence of SNVs (the first 3 columns with posterior estimates) and in the ABC-RF approach that considers the estimated recombination rate (designated as ‘FastSimBac’; the last 3 columns with posterior estimates) used to investigate the *S*. *pneumoniae* demographic history.** Presented are the median, 2.5% and 97.5% percentiles (%iles) of growth rates. S, number of segregating sites.
(PDF)

S3 Text Higher neutral genetic diversity in *S*. *mitis* than in *S*. *pneumoniae* is not due to differences in their mutation rate. **Fig (i). Experimental confirmation of comparable mutation rates between *S*. *mitis* and *S*. *pneumoniae*.** Three isolates per species per experiment were used, which were performed in biological triplicate. Error bars show SD from mean. **A** Spontaneous mutation rate for inhibitory concentration of Streptomycin (black) and Rifampicin (grey). No significant difference between species was identified (two-way ANOVA) for either Streptomycin (*P-value* = 0.36) or Rifampicin (*P-value* = 0.41). **B** Cell viability with and without UV exposure. Number of viable cells was not statistically significant at the 0.05 level between cells exposed to UV and those that were not. Significance between states tested by unpaired t-test (*P-value*: B2C2 = 0.38, C5T6 = 0.36, S1092G24C4 = 0.09, G54 = 0.08, D39 = 0.69, TIGR4 = 0.43). **C** Twenty-four-hour growth curves from starting OD600 of 0.002 (reaching 0.157–0.642) demonstrating comparable growth rate between species.
(PDF)

S1 Fig Pairwise SNV differences distribution for *S*. *mitis* and *S*. *pneumoniae*. **A.** Pairwise SNV differences distribution for *S*. *mitis* total sample (n = 119). **B.** Pairwise SNV differences distribution for *S*. *pneumoniae* total sample (n = 810). **C.** Pairwise SNV differences distribution between related hosts, between unrelated hosts and within host for *S*. *mitis*. **D.** Pairwise SNV differences distribution between unrelated hosts and within hosts for *S*. *pneumoniae* (for the African dataset, n = 230).
(PDF)

S2 Fig *S*. *pneumoniae* genetic diversity considering bigger thresholds of pairwise SNV differences to control for clonal relationships. The number of genes is plotted as a function of the number of genomes. Power law regression was fitted to the mean number of genes obtained across all permutations and for 1000 random samples of size 75 as in *S*. *mitis*. The parameterisation that best fitted the data was: Y = *a*X^*b*^ + *c*; **A.** Analysis of *S*. *pneumoniae* pangenomes for the isolates with a minimum of 1000 pairwise SNV differences (*b* = 0.16–0.32; mean = 0.23). Core genomes nucleotide diversity (*π*) estimates for Africa (Af), Asia (As) and European (Eu) samples: *π*_*Af*_ = 0.009; *π*_*As*_ = 0.010; *π*_*Eu*_ = 0.008. **B.** Analysis of *S*. *pneumoniae* pangenomes for the isolates with a minimum of 2000 pairwise SNV differences (*b* = 0.16–0.34; mean = 0.24. Core genomes nucleotide diversity estimates: *π*_*Af*_ = 0.009; *π*_*As*_ = 0.010; *π*_*Eu*_ = 0.008. **C.** Analysis of *S*. *pneumoniae* pangenomes for the isolates with a minimum of 2000 pairwise SNV differences (*b* = 0.19–0.31; mean = 0.28). Core genomes nucleotide diversity estimates: *π*_*Af*_ = 0.010; *π*_*As*_ = 0.011; *π*_*Eu*_ = 0.008. *S*. *mitis*, orange; *S*. *pneumoniae*, blue.
(PDF)

S3 Fig Population structure analyses for *S*. *pneumoniae* considering bigger thresholds of pairwise SNV differences to control for clonal relationships. **A&B.** PCA analysis of *S*. *pneumoniae* genetic variation for the isolates with a minimum of 1000 pairwise SNV differences (A, including serotype NT; B, excluding serotype NT). F_ST_s for Africa (Af), Asia (As) and European (Eu) population pairs: F_ST_(Af-As) = 0.0177 +/- 0.0027; F_ST_(Af-Eu) = 0.0146 +/- 0.0016; F_ST_(As-Eu) = 0.0310 +/- 0.0031. **C&D.** PCA analysis of *S*. *pneumoniae* genetic variation for the isolates with a minimum of 2000 pairwise SNV differences (C, including serotype NT; D, excluding serotype NT). F_ST_s: F_ST_(Af-As) = 0.0235 +/- 0.0027; F_ST_(Af-Eu) = 0.0069 +/- 0.0018; F_ST_(As-Eu) = 0.0253 +/- 0.0025. **E&F.** PCA analysis of *S*. *pneumoniae* genetic variation for the isolates with a minimum of 5600 pairwise SNV differences (E, including serotype NT; F, excluding serotype NT). F_ST_s: F_ST_(Af-As) = 0.0032 +/- 0.0018; F_ST_(Af-Eu) = -0.0088 +/- 0.0034; F_ST_(As-Eu) = 0.0042 +/- 0.0017. The NT cluster comprise nonencapsulated isolates which were previously reported to have higher recombination rates generating significantly more diversity within this cluster [44]. Colour code corresponds to geographic region: Africa, pink; Asia, blue; Europe, green. Abbreviations: pair diff, pairwise SNV differences; ST-NT, serotype NT.
(PDF)

S4 Fig Phylogenetic and population structure for *S*. *pneumoniae*’s total sample (including serotype NT). **A.** PCA computed in Plink v1.9 [93]. **B.** Maximum likelihood unrooted phylogenetic tree obtained using FastTree [84]. There are subclades of Asian lineages which belong exclusively to the PCA outlying serotypes NT (three subclades with isolates belonging mainly to GPSCs 28, 42, 60, 66, 118) and 19F (one subclade classified as GPSC-1), although both NT and 19F clades also include African and European lineages. The NT cluster comprise nonencapsulated isolates which were previously reported to have higher recombination rates generating significantly more diversity within this cluster [44]. Colour code corresponds to geographic region: Africa, pink; Asia, blue; Europe, green.
(PDF)

S5 Fig Distribution of p-values for whether genes have a higher ratio of number of non-synonymous variants to number of synonymous variants than a random set of simulated ratios for *S*. *mitis* (A) and *S*. *pneumoniae* (B). The horizontal line corresponds to the expected number of genes with p-values<0.05, under a null uniform distribution of p-values.
(PDF)

S6 Fig QQplots of the null *π*_*N*_/*π*_*S*_ distribution obtained from simulated 1Kb windows (wins) versus the observed *π*_*N*_/*π*_*S*_ distributions in genes from *S*. *mitis* (A) and *S*. *pneumoniae* (B). (PDF)

S1 Table Pangenome statistics for *S*. *pneumoniae* and *S*. *mitis*. Pangenome and core genome sizes units are number of genes. N, sample size. Regional (continental) random sample of unrelated isolates show means of 1000 random samples with size equal to that of the smaller regional sample size within each species. ‘All random sample of unrelated isolates’ in *S*. *pneumoniae* shows the mean of 1000 random samples with size equal to the observed *S*. *mitis* unrelated sample size.
(PDF)

S2 Table Between population divergence estimates (Hudson’s F_ST_ +/- SE). *S*. *mitis*, above diagonal; *S*. *pneumoniae*, below diagonal.
(PDF)

S3 Table Genome accession and geographical origin of *Streptococcus mitis* genomes. (PDF)
