# Supplementary material for: Long-term evolution of Streptococcus mitis and Streptococcus pneumoniae leads to higher genetic diversity within rather than between human populations
Source: PLoS Genet. 2024 Jun 6;20(6):e1011317. doi: 10.1371/journal.pgen.1011317 (PMC11185502; doi:10.1371/journal.pgen.1011317)
Supplement: S1 Text — Fig (i). Posterior predictive checks of the ABC approach implemented to investigate S. mitis demographic history. The density plots show, from left to right, the distribution of Tajima’s D obtained from simulating 1,500 250Kb-windows (blue) using as parameters the 25% percentile, the median, and the 75% percentile of estimated growth rates from across windows, and the corresponding to the observed Tajima’s D estimated across the 34 windows considered (Table A in S1 Text). Fig (ii). Posterior predictive checks of the ABC approach implemented to investigate S. pneumoniae demographic history. The density plots show, from left to right, the distribution of Tajima’s D obtained from simulating 1,500 250Kb-windows (blue) using as parameters the 25% percentile, the median, and the 75% percentile of estimated growth rates from across windows, and the corresponding to the observed Tajima’s D estimated across the 15 windows considered (Table B in S1 Text). Table A. Observed genetic diversity indices (S and Tajima’s D) and posterior estimates of growth rate obtained in the ABC-RF approach implemented to investigate S. mitis demographic history. Presented are the median, 2.5% and 97.5% percentiles of growth rates obtained from 30,000 simulations. S, number of segregating sites. Table B. Observed genetic diversity indices (S and Tajima’s D) and posterior estimates of growth rate obtained in the ABC-RF approach implemented to investigate S. pneumoniae demographic history. Presented are the median, 2.5% and 97.5% percentiles of growth rates obtained from 30,000 simulations. S, # of segregating sites. (PDF) [file pgen.1011317.s001.pdf]

S1 Text. Accuracy and model fit of the ABC approach used to evaluate *S. mitis* and *S. pneumoniae* demographic histories.

Parameter estimation with our ABC approach considering each SNV independently was very precise for both species. For the simulations with growth rate parameter of 0, the mean absolute error of the growth rate estimation was <0.001 for both species. For growth rate parameters >0, the NMAE when predicting the growth rate of a simulation was estimated as 0.041 for *S. pneumoniae* and as 0.03 for *S. mitis*.

Tajima's D calculated based on synonymous variation across core genomes was observed to vary more across windows in *S. pneumoniae* than that across windows in *S. mitis* (Tables A and B below). Nevertheless, graphical comparison of the posterior predictive checks allowed us to conclude that our approach recovers well the observed Tajima's D well for both species (Fig (i) and (ii) below). In *S. mitis*, the genetic diversity (Tajima's D) from an exponentially growing population with growth rate given by the 25% and 75% percentiles and median across simulated windows capture the range observed Tajima's D values. In *S. pneumoniae*, although the genetic diversity (Tajima's D) from an exponentially growing population with growth rates given by the median and 75% percentiles across simulated windows do not capture windows with bigger (less negative) Tajima's D values, the simulated genetic diversity distribution obtained with the growth rate given by the 25% percentile does capture the range observed Tajima's D almost fully. Thus, we conclude that an exponential growth model with a growth rate in the estimated range given by the window posterior medians describes the observed genetic diversity reasonably well, although the actual demography is likely more complex for *S. pneumoniae*.

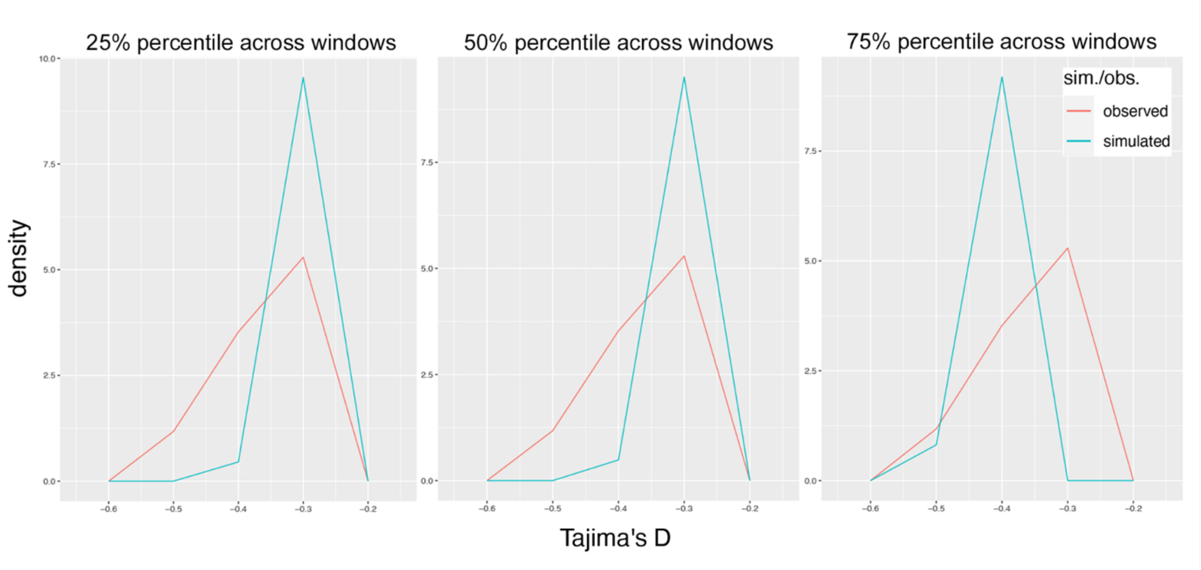

Fig (i). Posterior predictive checks of the ABC approach implemented to investigate *S.mitis* demographic history. The density plots show, from left to right, the distribution of Tajima's D obtained from simulating 1,500 250Kb-windows (blue)

using as parameters the 25% percentile, the median, and the 75% percentile of estimated growth rates from across windows, and the corresponding to the observed Tajima's D estimated across the 34 windows considered (Table A, below).

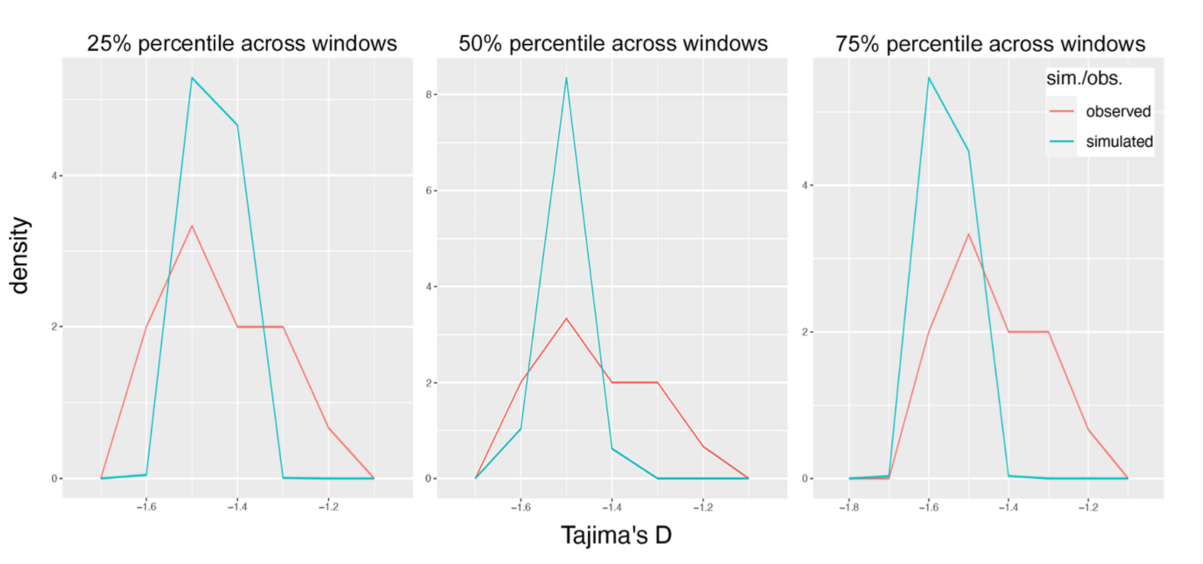

Fig (ii). Posterior predictive checks of the ABC approach implemented to investigate *S. pneumoniae* demographic history. The density plots show, from left to right, the distribution of Tajima's D obtained from simulating 1,500 250Kb-windows (blue) using as parameters the 25% percentile, the median, and the 75% percentile of estimated growth rates from across windows, and the corresponding to the observed Tajima's D estimated across the 15 windows considered (Table B, below).

Table A. Observed genetic diversity indices (S and Tajima's D) and posterior estimates of growth rate obtained in the ABC-RF approach implemented to investigate *S. mitis* demographic history. Presented are the median, 2.5% and 97.5% percentiles of growth rates obtained from 30,000 simulations. S, number of segregating sites.

Start of window	S	Tajima's D	Median posterior growth rate	2.5% Percentile posterior growth rate	97.5% Percentile posterior growth rate
0	19625	-0.3246	0.2	0.2	0.2
50000	20828	-0.3322	0.2	0.2	0.2
100000	21408	-0.4136	0.3	0.3	0.4
150000	21025	-0.3866	0.3	0.27	0.4
200000	19696	-0.4044	0.3	0.2	0.4
250000	19923	-0.3904	0.3	0.2	0.3
300000	18577	-0.4045	0.3	0.2	0.3
350000	19818	-0.3566	0.3	0.2	0.3
400000	21908	-0.3378	0.2	0.2	0.3
450000	21581	-0.3477	0.2	0.2	0.3
500000	22222	-0.3672	0.3	0.2	0.3
550000	24093	-0.3260	0.2	0.2	0.2
600000	23027	-0.3028	0.2	0.2	0.2
650000	22737	-0.3337	0.2	0.2	0.2
700000	23090	-0.2928	0.2	0.2	0.2
750000	22018	-0.2921	0.2	0.2	0.2
800000	21942	-0.2687	0.2	0.2	0.2
850000	23411	-0.3011	0.2	0.2	0.2
900000	23203	-0.2898	0.2	0.2	0.2
950000	24320	-0.3010	0.2	0.2	0.2
1000000	24662	-0.3367	0.2	0.2	0.2
1050000	22757	-0.3609	0.3	0.2	0.3
1100000	19447	-0.3601	0.2	0.2	0.2
1150000	19063	-0.3275	0.2	0.2	0.2
1200000	18088	-0.3480	0.3	0.2	0.3
1250000	20068	-0.3278	0.3	0.2	0.3
1300000	19847	-0.3514	0.3	0.2	0.3
1350000	18805	-0.3293	0.2	0.2	0.2
1400000	19910	-0.3656	0.3	0.2	0.3
1450000	20373	-0.4443	0.3	0.3	0.4
1500000	19342	-0.4534	0.3	0.3	0.4
1550000	18124	-0.4774	0.4	0.4	0.4
1600000	21706	-0.4520	0.3	0.3	0.4

1650000	22377	-0.4622	0.4	0.3	0.4

Table B. Observed genetic diversity indices (S and Tajima's D) and posterior estimates of growth rate obtained in the ABC-RF approach implemented to investigate *S. pneumoniae* demographic history. Presented are the median, 2.5% and 97.5% percentiles of growth rates obtained from 30,000 simulations. S, # of segregating sites.

Start of window	S	Tajima's D	Median posterior growth rate	2.5% Percentile posterior growth rate	97.5% Percentile posterior growth rate
0	9823	-1.5404	3.1	2.4	9.6
50000	8917	-1.5081	2.8	2.2	5.8
100000	9121	-1.5290	3.0	2.4	5.8
150000	10227	-1.6264	3.9	3.0	6.5
200000	9433	-1.5008	2.8	2.4	5.8
250000	8844	-1.4169	2.5	2.0	3.3
300000	9902	-1.3425	2.2	1.6	3.0
350000	9215	-1.3140	2.0	1.4	3.0
400000	9343	-1.3196	2.0	1.4	3.0
450000	9873	-1.4506	2.7	2.1	4.5
500000	10076	-1.5550	3.4	2.6	5.7
550000	9314	-1.6094	4.0	3.0	5.8
600000	10357	-1.3856	2.5	1.6	3.8
650000	9383	-1.3840	2.5	1.6	3.7
700000	7733	-1.2439	1.9	1.4	2.8
