# Supplementary material for: Long-term evolution of Streptococcus mitis and Streptococcus pneumoniae leads to higher genetic diversity within rather than between human populations
Source: PLoS Genet. 2024 Jun 6;20(6):e1011317. doi: 10.1371/journal.pgen.1011317 (PMC11185502; doi:10.1371/journal.pgen.1011317)
Supplement: S2 Text — Fig (i). Comparison between the distribution of observed number of segregating sites (pink; across 83 S. pneumoniae core genome windows) and the distribution of simulated number of segregating sites used in the ABC approach implemented to investigate S. pneumoniae demographic history. The distribution of simulated S values follows the observed distribution closely. S, number of segregating sites. Table A. Observed genetic diversity indices (S and Tajima’s D) and posterior estimates of growth rate obtained in the ABC-RF approach that considers all independence of SNVs (the first 3 columns with posterior estimates) and in the ABC-RF approach that considers the estimated recombination rate (designated as ‘FastSimBac’; the last 3 columns with posterior estimates) used to investigate the S. pneumoniae demographic history. Presented are the median, 2.5% and 97.5% percentiles (%iles) of growth rates. S, number of segregating sites. (PDF) [file pgen.1011317.s002.pdf]

S2 Text. Assessing the influence of recombination in the estimation of growth rates for *S. pneumoniae* via the ABC approach in FastSimBac

As *S. pneumoniae* shows higher (neutral) LD values at distances < 1500 bps, we evaluated if recombination has any influence in the model fit and estimation of growth rates in an ABC model of exponential growth. To this end, the coalescent simulator FastSimBac [1] was used to generate sequences under an exponential growth model, which incorporated mutation and recombination. Due to computational constrains, we simulated 100Kb segments with sample size and numbers of SNVs matching the ones estimated for overlapping sliding windows of the same size across *S. pneumoniae* sampled genomes with an offset of 10 Kb between adjacent windows.

In each simulation, parameters were randomly drawn from the following priors. Exponential growth rate was taken from an exponential distribution with rate 0.05. Mutation rate was set in each run by drawing a number of segregating sites S from a uniform distribution with boundaries selected at random from the observed number of segregating sites estimated in 100Kb-sized windows across *S. pneumoniae* sampled genomes, and scaling S with the mean number of segregating sites obtained from additional three FastSimBac runs with the same growth rate, recombination rate drawn from the prior described above, and a mutation rate of 1. Under a coalescence framework, the mean number of segregating sites obtained from these three simulations gives us a rough estimate of the expected length of the whole genealogy, which can then be used to properly scale the mutation rate. We only performed three simulations due to computational constrains, however we sought that these were sufficient to average out the variation in the independent estimates, since the number of segregating sites simulated across simulations was similar to the observed ones (Fig (i) below). Effective recombination rate was taken both from a gamma distribution with shape parameter 1.12 and rate parameter 8 (25,500 simulations) and from a gamma distribution with shape parameter 5.6 and rate parameter 40 (5,600 simulations). Both distributions were truncated at a recombination rate of 0.3, due to computational constraints). Computational constraints restricted us from running models with higher scaled recombination rates (which in FastSimBac is in units of $2N_e$; FastSimBac then internally halves this value to scale the simulated the genealogy or Ancestral Recombination Graph to $4N_e$). We then ran an identical ABC-RF analysis as in our main simulation tool. The NMAE of the posterior growth rates was <0.15, attesting to the accuracy of the random-forest classifier.

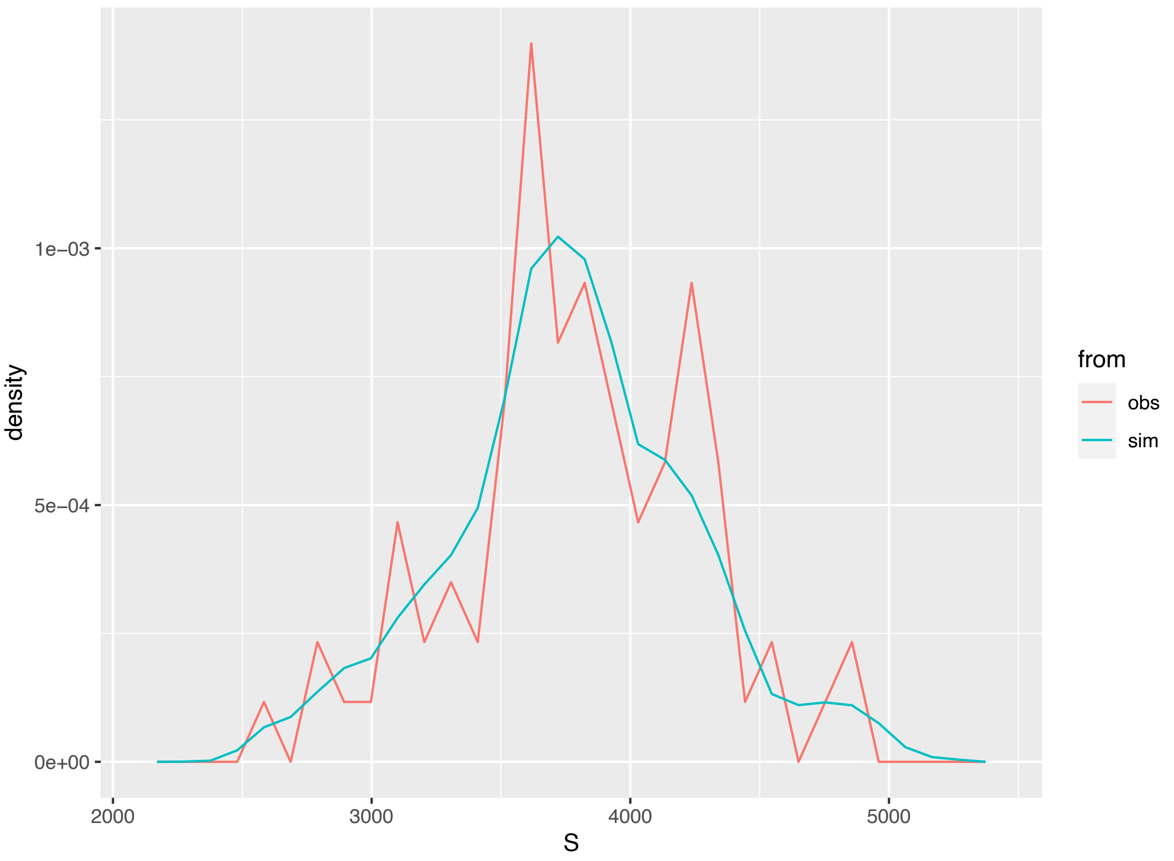

Fig (i). Comparison between the distribution of observed number of segregating sites (pink; across 83 *S. pneumoniae* core genome windows) and the distribution of simulated number of segregating sites used in the ABC approach implemented to investigate *S. pneumoniae* demographic history. The distribution of simulated S values follows the observed distribution closely. S, number of segregating sites.

As we estimated a recombination rate of 0.28 in units of $2N_e$ (based on our mcorr estimate of 0.14 in units of N_e; Table 1 in the main text), we extracted all simulations with a scaled recombination rate between 0.2 and 0.3, resulting in 4,368 simulations. Growth rate estimates per window were comparable between the two approaches (median absolute difference 0.11, standard deviation 0.39; Table A below), which validated our approach.

Table A. Observed genetic diversity indices (S and Tajima's D) and posterior estimates of growth rate obtained in the ABC-RF approach that considers all independence of SNVs (the first 3 columns with posterior estimates) and in the ABC-RF approach that considers the estimated recombination rate (designated as 'FastSimBac'; the last 3 columns with posterior estimates) used to investigate the *S. pneumoniae* demographic history. Presented are the median, 2.5% and 97.5% percentiles (%iles) of growth rates. S, number of segregating sites.

Start of window	S	Tajima's D	Median posterior growth rate	2.5 %ile posterior growth rate	97.5 %ile posterior growth rate	FastSimBac Median posterior growth rate	FastSimBac 2.5 %ile posterior growth rate	FastSimBac 97.5 %ile posterior growth rate
0	4259	-1.6879	4.4	3.6	6.7	4.5	3.6	8.0
10000	3864	-1.5523	3.2	2.6	4.6	3.3	2.4	4.2
20000	3633	-1.5004	2.7	2.0	3.8	2.6	2.2	3.8
30000	3195	-1.4573	2.4	1.9	3.8	2.6	2.0	3.6
40000	3302	-1.2706	1.8	1.3	2.9	1.7	1.2	2.8
50000	2822	-1.1282	1.2	1.0	1.8	1.2	0.8	1.8
60000	2606	-0.9952	1.1	0.7	1.4	1.0	0.6	1.4
70000	2998	-1.1193	1.2	1.0	2.0	1.2	0.8	1.8
80000	3083	-1.2466	1.7	1.1	2.9	1.5	1.1	2.8
90000	3476	-1.3296	2.1	1.4	5.4	2.3	1.4	3.9
100000	3339	-1.3801	2.4	1.6	6.3	2.6	1.5	5.2
110000	3288	-1.4102	2.6	1.7	6.9	2.6	1.5	8.0
120000	3615	-1.4789	2.9	1.9	11.4	2.8	1.7	8.0
130000	4162	-1.4128	2.6	1.6	11.4	2.6	1.6	8.0
140000	3909	-1.5852	4.1	2.6	11.8	3.9	2.3	10.3
150000	4519	-1.6263	6.2	2.8	14.8	4.1	2.6	11.5
160000	4848	-1.7094	6.5	3.1	14.8	5.1	3.1	11.5
170000	4850	-1.6603	4.4	3.0	11.8	4.3	3.1	10.3
180000	4780	-1.6457	4.3	2.8	11.4	4.3	3.1	9.8
190000	4176	-1.6238	4.1	2.8	11.4	3.9	2.7	8.0
200000	3801	-1.6383	4.0	2.8	11.4	3.9	2.9	8.0
210000	4223	-1.6034	3.7	2.8	5.8	3.8	2.7	5.2
220000	4148	-1.5180	2.8	2.1	3.9	2.8	2.3	3.9
230000	3609	-1.5975	3.7	2.9	4.6	3.6	2.7	4.4
240000	3462	-1.6758	3.8	3.4	4.9	4.2	3.5	5.1
250000	3557	-1.6878	4.0	3.0	4.8	4.2	3.5	5.1
260000	2818	-1.5830	3.3	2.7	4.1	3.2	2.4	4.0
270000	2906	-1.5956	3.6	2.7	4.6	3.6	2.6	4.5
280000	3144	-1.6751	4.1	3.5	5.0	4.1	3.5	5.5
290000	3613	-1.6440	3.8	3.2	4.9	3.9	3.3	5.1

300000	4132	-1.5412	3.2	2.7	4.1	3.0	2.4	3.9
310000	3738	-1.3988	2.6	1.9	4.1	2.6	1.7	3.6
320000	3635	-1.4244	2.6	1.9	4.1	2.6	1.8	3.9
330000	3855	-1.4278	2.6	1.9	4.1	2.6	1.7	3.9
340000	3955	-1.3489	2.3	1.3	5.4	2.4	1.3	4.2
350000	3651	-1.3201	2.1	1.2	3.8	2.2	1.2	3.4
360000	3658	-1.3185	2.1	1.2	3.8	2.2	1.2	3.4
370000	3476	-1.2944	2.1	1.2	3.1	1.9	1.2	3.4
380000	3383	-1.0431	1.2	0.7	1.9	1.1	0.7	1.8
390000	3475	-1.0352	1.2	0.6	2.9	1.2	0.6	2.4
400000	3136	-1.0359	1.2	0.6	2.9	1.2	0.6	2.4
410000	3248	-1.0807	1.2	0.7	2.9	1.2	0.7	2.8
420000	3665	-1.1417	1.4	0.8	2.9	1.3	0.9	2.8
430000	3945	-1.2524	2.1	1.0	3.9	1.7	1.1	3.8
440000	4059	-1.3447	2.3	1.6	3.9	2.4	1.5	3.8
450000	4270	-1.2563	2.0	1.1	2.9	1.8	1.2	3.1
460000	4436	-1.2694	2.1	1.2	2.9	1.9	1.2	3.2
470000	4232	-1.1993	1.7	1.0	2.9	1.5	1.0	2.8
480000	3845	-1.2940	2.1	1.3	2.9	1.9	1.2	3.1
490000	3897	-1.3615	2.1	1.6	2.9	2.3	1.6	3.3
500000	3928	-1.4210	2.3	1.9	3.0	2.5	1.8	3.4
510000	4367	-1.5721	3.4	2.7	4.6	3.2	2.4	4.1
520000	4223	-1.5823	3.7	2.8	5.8	3.6	2.4	5.2
530000	3823	-1.5159	2.8	2.1	5.4	3.1	2.3	4.4
540000	3570	-1.4546	2.6	1.9	6.0	2.6	1.8	4.2
550000	3573	-1.5123	2.8	2.1	5.4	3.0	2.3	4.4
560000	3771	-1.4945	2.9	2.1	5.8	3.2	2.1	5.2
570000	4015	-1.5786	3.8	2.9	6.2	3.7	2.6	5.4
580000	4313	-1.5901	3.9	3.0	6.2	3.8	2.6	5.4
590000	3984	-1.5801	4.1	2.8	6.2	3.8	2.4	5.4
600000	4309	-1.6318	4.9	3.0	7.7	4.2	2.9	8.0
610000	3756	-1.5532	3.7	2.6	6.2	3.7	2.4	5.4
620000	3599	-1.5032	3.1	2.1	5.8	3.4	2.0	5.2
630000	3773	-1.5251	3.2	2.2	5.5	3.5	2.4	5.2
640000	3920	-1.6262	4.6	3.0	6.8	4.0	3.0	6.0
650000	3869	-1.7023	6.0	3.7	8.3	5.1	3.3	8.0
660000	3736	-1.7574	6.2	4.4	8.4	5.6	4.0	8.0
670000	3553	-1.7753	6.3	4.6	8.5	5.6	4.2	7.8
680000	3764	-1.8158	6.6	5.1	8.9	6.0	5.0	8.1
690000	3756	-1.6928	5.3	3.5	7.1	5.1	3.3	7.4
700000	3711	-1.6255	4.0	3.2	7.0	3.9	2.9	6.5

710000	3834	-1.6450	4.4	3.2	7.0	4.2	3.3	7.2
720000	4116	-1.4818	3.2	2.0	5.2	3.3	1.9	5.4
730000	4257	-1.3479	2.6	1.7	4.2	2.7	1.6	4.6
740000	4376	-1.1844	1.6	1.0	2.9	1.6	1.0	3.6
750000	4209	-1.0134	1.1	0.7	2.4	1.2	0.7	1.7
760000	4362	-1.0196	1.1	0.7	2.4	1.2	0.7	1.7
770000	4500	-0.9805	1.1	0.5	1.7	1.0	0.6	1.7
780000	4255	-0.9402	1.0	0.5	1.7	0.9	0.5	1.6
790000	4072	-1.0265	1.2	0.9	2.0	1.2	0.7	1.7
800000	3642	-0.9338	1.0	0.5	1.6	0.9	0.6	1.5
810000	3469	-0.8529	0.8	0.5	1.5	0.8	0.4	1.3
820000	3102	-0.9740	1.1	0.6	1.6	1.0	0.6	1.5
830000	2679	-1.0597	1.2	0.9	2.4	1.2	0.7	1.9

Supplementary Reference

1. De Maio N, Wilson DJ. The Bacterial Sequential Markov Coalescent. Genetics. 2017;206(1):333-43. Epub 20170303. doi: 10.1534/genetics.116.198796. PubMed PMID: 28258183; PubMed Central PMCID: PMCPMC5419479.
