# Supplementary material for: Long-term evolution of Streptococcus mitis and Streptococcus pneumoniae leads to higher genetic diversity within rather than between human populations
Source: PLoS Genet. 2024 Jun 6;20(6):e1011317. doi: 10.1371/journal.pgen.1011317 (PMC11185502; doi:10.1371/journal.pgen.1011317)
Supplement: S3 Text — Fig (i). Experimental confirmation of comparable mutation rates between S. mitis and S. pneumoniae. Three isolates per species per experiment were used, which were performed in biological triplicate. Error bars show SD from mean. A Spontaneous mutation rate for inhibitory concentration of Streptomycin (black) and Rifampicin (grey). No significant difference between species was identified (two-way ANOVA) for either Streptomycin (P-value = 0.36) or Rifampicin (P-value = 0.41). B Cell viability with and without UV exposure. Number of viable cells was not statistically significant at the 0.05 level between cells exposed to UV and those that were not. Significance between states tested by unpaired t-test (P-value: B2C2 = 0.38, C5T6 = 0.36, S1092G24C4 = 0.09, G54 = 0.08, D39 = 0.69, TIGR4 = 0.43). C Twenty-four-hour growth curves from starting OD600 of 0.002 (reaching 0.157–0.642) demonstrating comparable growth rate between species. (PDF) [file pgen.1011317.s003.pdf]

S3 Text. Higher neutral genetic diversity in *S. mitis* than in *S. pneumoniae* is not due to differences in their mutation rate

We confirmed experimentally and bioinformatically that the observed differences in neutral genetic diversity between *S. mitis* and *S. pneumoniae* are not due to differences in mutation rate between the species.

Firstly, we compared experimental determinations of spontaneous mutation rates for antibiotic resistant markers between the two species. Cells were grown to mid-exponential phase in liquid culture for 18 hours at 37°C, 5% CO_2. Three strains from the *S. mitis* dataset (one from each host population) were randomly selected and tested along with three laboratory pneumococcal strains (G54, D39 or TIGR4) in biological triplicate. Minimum inhibitory concentrations of Rifampicin and Streptomycin were determined via 10-fold serial dilutions of antibiotic for each strain under investigation to ensure a suitably high level of antibiotic was used (i.e., that the bacterium did not have pre-existing resistance). Concentrated cultures (OD600 = 0.1) were plated on 32μg/ml Rifampicin and 500μg/ml Streptomycin in triplicate on BHI agar, 3% defibrinated horse blood (Oxoid) plates and grown for 24 hours at 37°C, 5% CO2. Following incubation, discrete colonies were counted. For normalisation across strains, mutation rate (μ) was calculated per cell division from the mean number of (normally distributed) colonies counted across repeats. This calculation was under the assumptions that mutations arise throughout the cell cycle and cells are grown in an asynchronous population [1, 2]:

$$\mu = \frac{ln2m}{N_t - 1}$$

Where, m = mean number of mutations per culture (i.e., number of colonies)

Nt = total number of starting cells

Resistance to both antibiotics can be accomplished through a singular point mutation. For both antibiotics, all *S. mitis* (and *S. pneumoniae*) strains generated discrete colonies, indicating occurrence of point mutations and a functional DNA mismatch repair system (as known for the *S. pneumoniae* strains used). Spontaneous mutation rates between antibiotics and between strains did not differ significantly (Fig (i)A, below).

Secondly, we evaluated the effects of UV-exposure on cell viability in both species. Concentrated liquid cultures (OD600 = 0.1) of each of the three *S. mitis* and three *S. pneumoniae* strains were grown in 50ml BHI (Oxoid). Cultures were centrifuged at 4000rpm for 10 minutes to pellet cells, which were resuspended in 20ml 0.9% NaCl. Half of the resuspension (depth of resuspension in petri dish = 3.75mm) was exposed to a UV radiation source producing 10 J/min for 45 seconds. 10-fold serial dilutions (up to 10^{-8}) of exposed and non-exposed culture were pipetted onto BHI, 3% horse blood agar plates in triplicate and incubated for 24 hours at 37°C, 5% CO2 [3].

Following incubation, discrete colonies were counted and colony forming units per ml (CFU/ml) were calculated.
After UV exposure, discrete colonies were identified across all six strains, and the number of CFU/ml was not significantly different between exposure/non exposure states. This confirms effective DNA nucleotide excision repair removing pyrimidine dimers generated by UVR exposure, which allows DNA replication to occur and to bacterial growth to ensue (Fig (i)B, below).

Thirdly, DNA repair during DNA replication was assessed by analysing the genetic variation of the *polA* gene, which encodes DNA polymerase I. This gene is highly variable, although a set of conserved residues have previously been proven essential for function [4]. All of these residues were ubiquitously present across isolates of both the *S. mitis* and *S. pneumoniae* datasets.

Finally, growth rate between species was experimentally compared. Liquid cultures were grown in BHI for 18 hours at 37°C, 5% CO2 and OD600 measurements were taken every hour for 24 hours.
An increased rate of DNA replication (faster growth) results in an accruement of genetic variation because of the inherent error rate of DNA replication (despite the proofreading capability of DNA polymerases). Thus, a higher rate of division accumulates more genetic variation. However, under laboratory conditions, the growth rate of *S. mitis* was comparable with *S. pneumoniae* (Fig (i)C, below). Inherently, the doubling times were similar between the species, most likely reflecting similar generation times in natural conditions.

In combination, these results support *S. mitis* to generate de novo mutation at a comparable rate to that of *S. pneumoniae*, and therefore it cannot explain the differences in genetic diversity observed between species. Since it has been shown that spontaneous mutation rates for antibiotic resistance are consistently higher than mutation rates from mutation accumulation experiments with whole genome sequencing [5], we use a phylogenetic estimate determined for *S. pneumoniae* in our modelling of the population history of *S. mitis* and *S pneumoniae* [6]. A phylogenetic estimate of mutation rate allows to account for the generation time of these organisms in their natural environment.

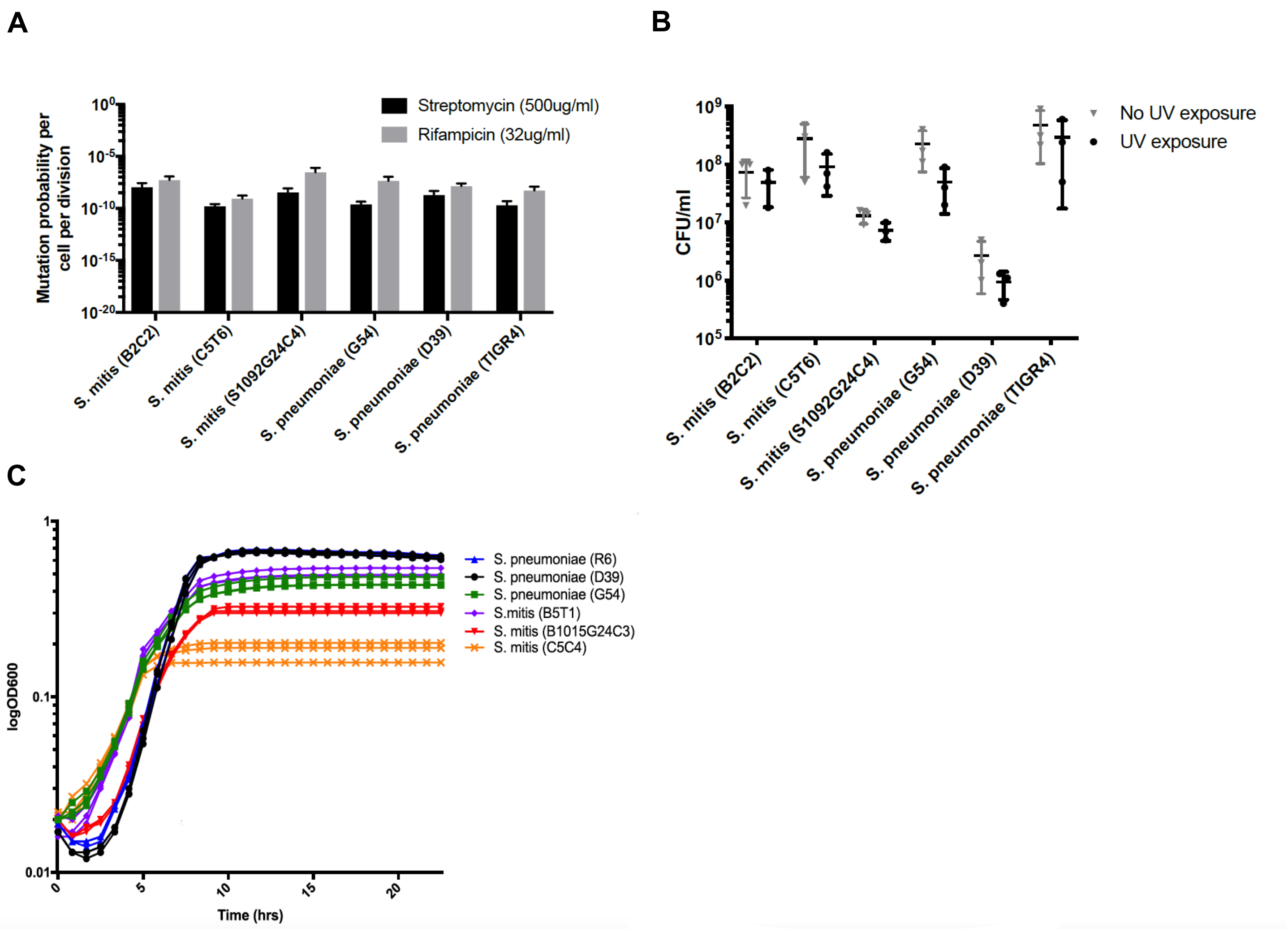

Fig (i). Experimental confirmation of comparable mutation rates between *S. mitis* and *S. pneumoniae*. Three isolates per species per experiment were used, which were performed in biological triplicate. Error bars show SD from mean. **A** Spontaneous mutation rate for inhibitory concentration of Streptomycin (black) and Rifampicin (grey). No significant difference between species was identified (two-way ANOVA) for either Streptomycin (*P-value* = 0.36) or Rifampicin (*P-value* = 0.41). **B** Cell viability with and without UV exposure. Number of viable cells was not statistically significant at the 0.05 level between cells exposed to UV and those that were not. Significance between states tested by unpaired t-test (*P-value*: B2C2=0.38, C5T6=0.36, S1092G24C4=0.09, G54=0.08, D39=0.69, TIGR4=0.43). **C** Twenty-four-hour growth curves from starting OD600 of 0.002 (reaching 0.157-0.642) demonstrating comparable growth rate between species.

Supplementary References

1. Lea DE, Coulson CA. The distribution of the numbers of mutants in bacterial populations. J Genet. 1949;49(3):264-85. doi: 10.1007/BF02986080. PubMed PMID: 24536673.
2. Foster PL. Methods for determining spontaneous mutation rates. Method Enzymol. 2006;409:195-213. doi: 10.1016/S0076-6879(05)09012-9. PubMed Central PMID: WOS:000238354800012.
3. De Ste Croix M. The Type I Restriction Modification System Spn III of Streptococcus pneumoniae[dissertation]. Leicester, UK: University of Leicester; 2017.
4. Patel PH, Loeb LA. DNA polymerase active site is highly mutable: Evolutionary consequences. P Natl Acad Sci USA. 2000;97(10):5095-100. doi: DOI 10.1073/pnas.97.10.5095. PubMed PMID: WOS:000086998500019.
5. Lee H, Popodi E, Tang H, Foster PL. Rate and molecular spectrum of spontaneous mutations in the bacterium *Escherichia coli* as determined by whole-genome sequencing. Proc Natl Acad Sci U S A. 2012;109(41):E2774-83. Epub 20120918. doi: 10.1073/pnas.1210309109. PubMed PMID: 22991466; PubMed Central PMCID: PMCPMC3478608.
6. Croucher NJ, Harris SR, Fraser C, Quail MA, Burton J, van der Linden M, et al. Rapid pneumococcal evolution in response to clinical interventions. Science. 2011;331(6016):430-4. doi: 10.1126/science.1198545. PubMed PMID: 21273480; PubMed Central PMCID: PMCPMC3648787.
