# Supplementary material for: Long-term evolution of Streptococcus mitis and Streptococcus pneumoniae leads to higher genetic diversity within rather than between human populations
Source: PLoS Genet. 2024 Jun 6;20(6):e1011317. doi: 10.1371/journal.pgen.1011317 (PMC11185502; doi:10.1371/journal.pgen.1011317)
Supplement: S1 Fig — A. Pairwise SNV differences distribution for S. mitis total sample (n = 119). B. Pairwise SNV differences distribution for S. pneumoniae total sample (n = 810). C. Pairwise SNV differences distribution between related hosts, between unrelated hosts and within host for S. mitis. D. Pairwise SNV differences distribution between unrelated hosts and within hosts for S. pneumoniae (for the African dataset, n = 230). (PDF) [file pgen.1011317.s004.pdf]

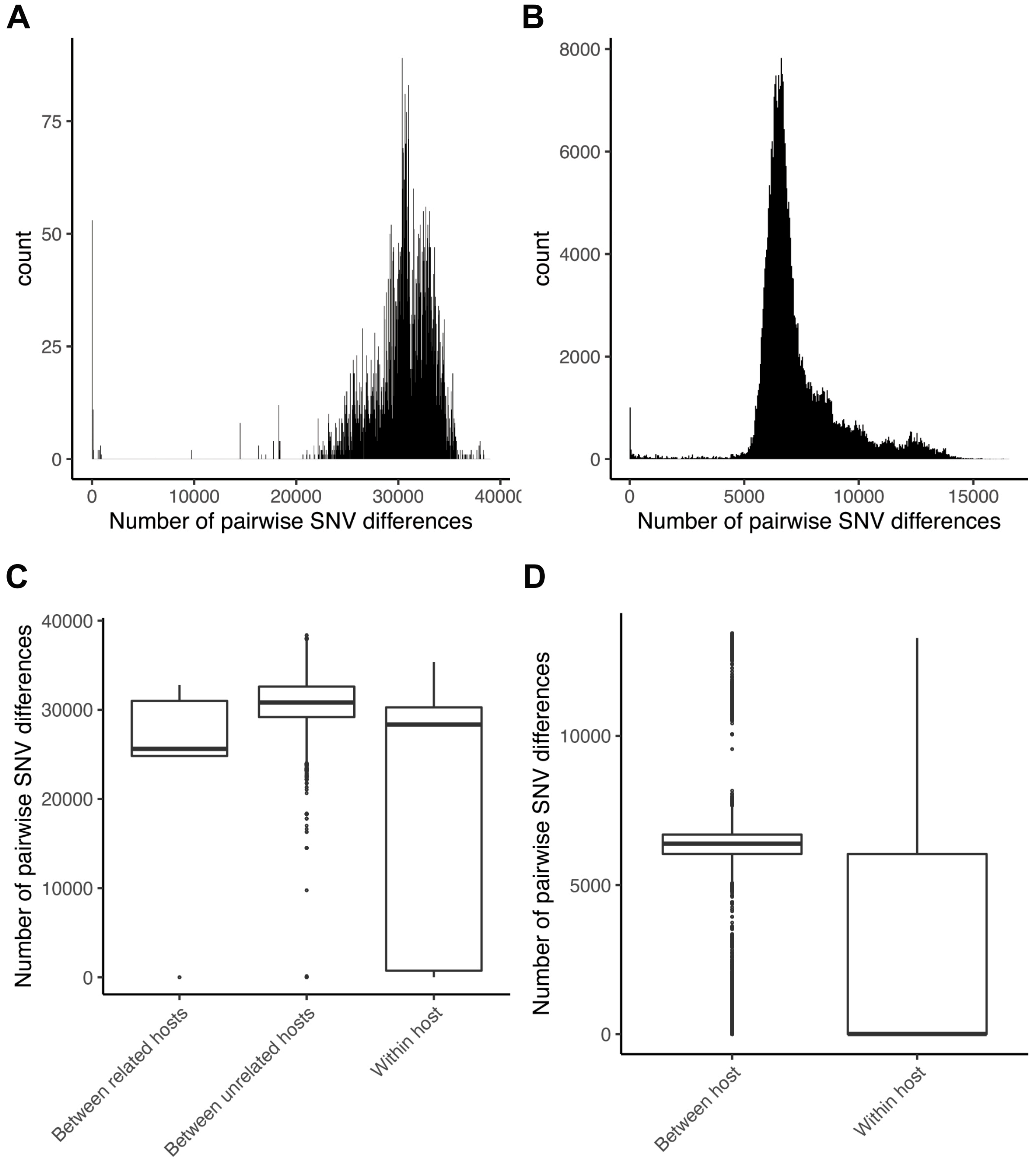

S1 Fig. Pairwise SNV differences distribution for *S. mitis* and *S. pneumoniae*. A. Pairwise SNV differences distribution for *S. mitis* total sample (n=119). **B.** Pairwise SNV differences distribution for *S. pneumoniae* total sample (n=810). **C.** Pairwise SNV differences distribution between related hosts, between unrelated hosts and within host for *S. mitis*. **D.** Pairwise SNV differences distribution between unrelated hosts and within hosts for *S. pneumoniae (*for the African dataset, n=230).
