# Supplementary material for: Long-term evolution of Streptococcus mitis and Streptococcus pneumoniae leads to higher genetic diversity within rather than between human populations
Source: PLoS Genet. 2024 Jun 6;20(6):e1011317. doi: 10.1371/journal.pgen.1011317 (PMC11185502; doi:10.1371/journal.pgen.1011317)
Supplement: S2 Fig — The number of genes is plotted as a function of the number of genomes. Power law regression was fitted to the mean number of genes obtained across all permutations and for 1000 random samples of size 75 as in S. mitis. The parameterisation that best fitted the data was: Y = aXb + c; A. Analysis of S. pneumoniae pangenomes for the isolates with a minimum of 1000 pairwise SNV differences (b = 0.16–0.32; mean = 0.23). Core genomes nucleotide diversity (π) estimates for Africa (Af), Asia (As) and European (Eu) samples: πAf = 0.009; πAs = 0.010; πEu = 0.008. B. Analysis of S. pneumoniae pangenomes for the isolates with a minimum of 2000 pairwise SNV differences (b = 0.16–0.34; mean = 0.24. Core genomes nucleotide diversity estimates: πAf = 0.009; πAs = 0.010; πEu = 0.008. C. Analysis of S. pneumoniae pangenomes for the isolates with a minimum of 2000 pairwise SNV differences (b = 0.19–0.31; mean = 0.28). Core genomes nucleotide diversity estimates: πAf = 0.010; πAs = 0.011; πEu = 0.008. S. mitis, orange; S. pneumoniae, blue. (PDF) [file pgen.1011317.s005.pdf]

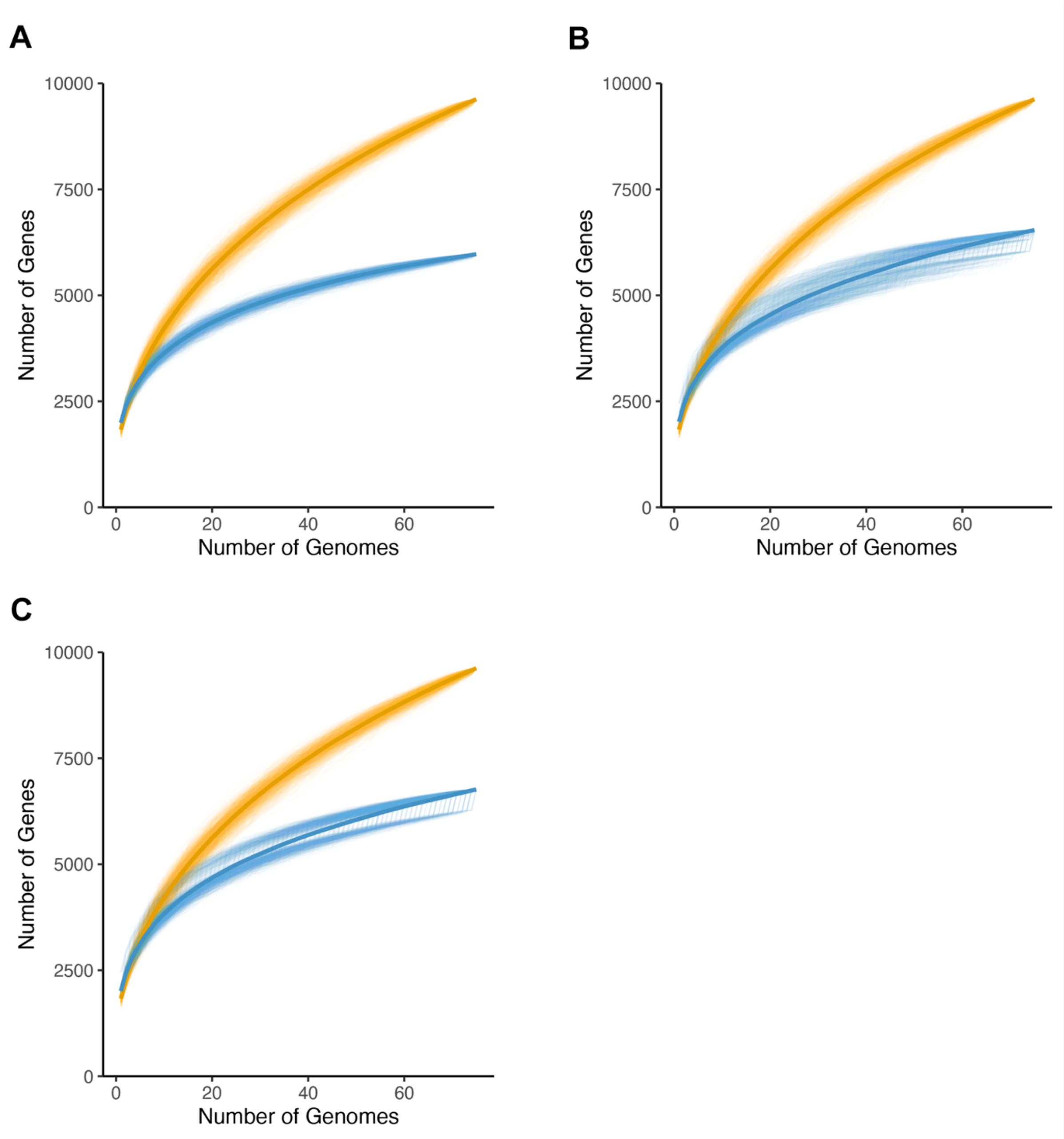

S2 Fig. *S. pneumoniae* genetic diversity considering bigger thresholds of pairwise SNV differences to control for clonal relationships. The number of genes is plotted as a function of the number of genomes. Power law regression was fitted to the mean number of genes obtained across all permutations and for 1000 random samples of size 75 as in *S. mitis*. The parameterisation that best fitted the data was: $Y = aX^b + c$; **A.** Analysis of *S. pneumoniae* pangenomes for the isolates with a minimum of 1000 pairwise SNV differences (b = 0.16 – 0.32; mean=0.23). Core genomes nucleotide diversity (π) estimates for Africa (Af), Asia (As) and European (Eu) samples: π_{Af}= 0.009; π_{As}= 0.010; π_{Eu}= 0.008. **B.** Analysis of *S. pneumoniae* pangenomes for the isolates with a minimum of 2000 pairwise SNV differences (b = 0.16 – 0.34; mean=0.24. Core genomes nucleotide diversity estimates: π_{Af}= 0.009; π_{As}= 0.010; π_{Eu}= 0.008. **C.** Analysis of *S. pneumoniae* pangenomes for the isolates with a minimum of 2000 pairwise SNV

differences (b = 0.19 – 0.31; mean=0.28). Core genomes nucleotide diversity estimates: π_{Af}= 0.010; π_{As}= 0.011; π_{Eu}= 0.008. *S. mitis*, orange; *S. pneumoniae*, blue.
