# Supplementary material for: Long-term evolution of Streptococcus mitis and Streptococcus pneumoniae leads to higher genetic diversity within rather than between human populations
Source: PLoS Genet. 2024 Jun 6;20(6):e1011317. doi: 10.1371/journal.pgen.1011317 (PMC11185502; doi:10.1371/journal.pgen.1011317)
Supplement: S3 Fig — A&B. PCA analysis of S. pneumoniae genetic variation for the isolates with a minimum of 1000 pairwise SNV differences (A, including serotype NT; B, excluding serotype NT). FSTs for Africa (Af), Asia (As) and European (Eu) population pairs: FST(Af-As) = 0.0177 +/- 0.0027; FST(Af-Eu) = 0.0146 +/- 0.0016; FST(As-Eu) = 0.0310 +/- 0.0031. C&D. PCA analysis of S. pneumoniae genetic variation for the isolates with a minimum of 2000 pairwise SNV differences (C, including serotype NT; D, excluding serotype NT). FSTs: FST(Af-As) = 0.0235 +/- 0.0027; FST(Af-Eu) = 0.0069 +/- 0.0018; FST(As-Eu) = 0.0253 +/- 0.0025. E&F. PCA analysis of S. pneumoniae genetic variation for the isolates with a minimum of 5600 pairwise SNV differences (E, including serotype NT; F, excluding serotype NT). FSTs: FST(Af-As) = 0.0032 +/- 0.0018; FST(Af-Eu) = -0.0088 +/- 0.0034; FST(As-Eu) = 0.0042 +/- 0.0017. The NT cluster comprise nonencapsulated isolates which were previously reported to have higher recombination rates generating significantly more diversity within this cluster [44]. Colour code corresponds to geographic region: Africa, pink; Asia, blue; Europe, green. Abbreviations: pair diff, pairwise SNV differences; ST-NT, serotype NT. (PDF) [file pgen.1011317.s006.pdf]

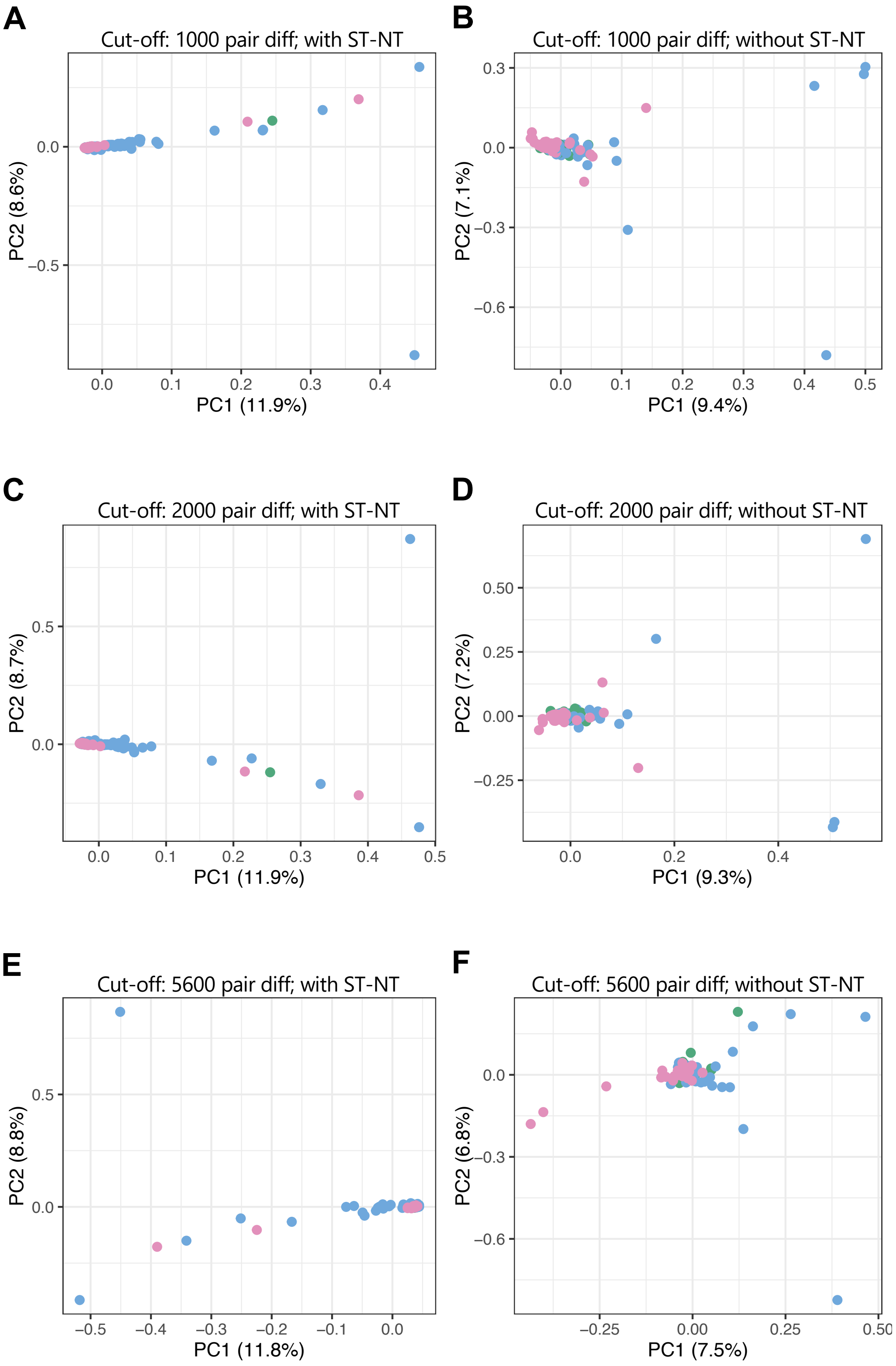

S3 Fig. Population structure analyses for *S. pneumoniae* considering bigger thresholds of pairwise SNV differences to control for clonal relationships. A&B. PCA analysis of *S. pneumoniae* genetic variation for the isolates with a minimum of 1000 pairwise SNV differences (A, including serotype NT; B, excluding serotype NT). F_{ST}s for Africa (Af), Asia (As) and European (Eu) population pairs: F_{ST}(Af-As)= 0.0177

+/- 0.0027; F_{ST}(Af-Eu)= 0.0146 +/- 0.0016; F_{ST}(As-Eu)= 0.0310 +/- 0.0031. **C&D.** PCA analysis of *S. pneumoniae* genetic variation for the isolates with a minimum of 2000 pairwise SNV differences (C, including serotype NT; D, excluding serotype NT). F_{ST}s: F_{ST}(Af-As)= 0.0235 +/- 0.0027; F_{ST}(Af-Eu)= 0.0069 +/- 0.0018; F_{ST}(As-Eu)= 0.0253 +/- 0.0025. **E&F.** PCA analysis of *S. pneumoniae* genetic variation for the isolates with a minimum of 5600 pairwise SNV differences (E, including serotype NT; F, excluding serotype NT). F_{ST}s: F_{ST}(Af-As)= 0.0032 +/- 0.0018; F_{ST}(Af-Eu)= -0.0088 +/- 0.0034; F_{ST}(As-Eu)= 0.0042 +/- 0.0017. The NT cluster comprise nonencapsulated isolates which were previously reported to have higher recombination rates generating significantly more diversity within this cluster [1]. Colour code corresponds to geographic region: Africa, pink; Asia, blue; Europe, green. Abbreviations: pair diff, pairwise SNV differences; ST-NT, serotype NT.

Supplementary Reference

[1] Chewapreecha C, Harris SR, Croucher NJ, Turner C, Marttinen P, Cheng L, et al. Dense genomic sampling identifies highways of pneumococcal recombination. Nat Genet. 2014;46(3):305-+. doi: 10.1038/ng.2895. PubMed PMID: WOS:000332036700016.
