# Supplementary material for: Long-term evolution of Streptococcus mitis and Streptococcus pneumoniae leads to higher genetic diversity within rather than between human populations
Source: PLoS Genet. 2024 Jun 6;20(6):e1011317. doi: 10.1371/journal.pgen.1011317 (PMC11185502; doi:10.1371/journal.pgen.1011317)
Supplement: S4 Fig — A. PCA computed in Plink v1.9 [93]. B. Maximum likelihood unrooted phylogenetic tree obtained using FastTree [84]. There are subclades of Asian lineages which belong exclusively to the PCA outlying serotypes NT (three subclades with isolates belonging mainly to GPSCs 28, 42, 60, 66, 118) and 19F (one subclade classified as GPSC-1), although both NT and 19F clades also include African and European lineages. The NT cluster comprise nonencapsulated isolates which were previously reported to have higher recombination rates generating significantly more diversity within this cluster [44]. Colour code corresponds to geographic region: Africa, pink; Asia, blue; Europe, green. (PDF) [file pgen.1011317.s007.pdf]

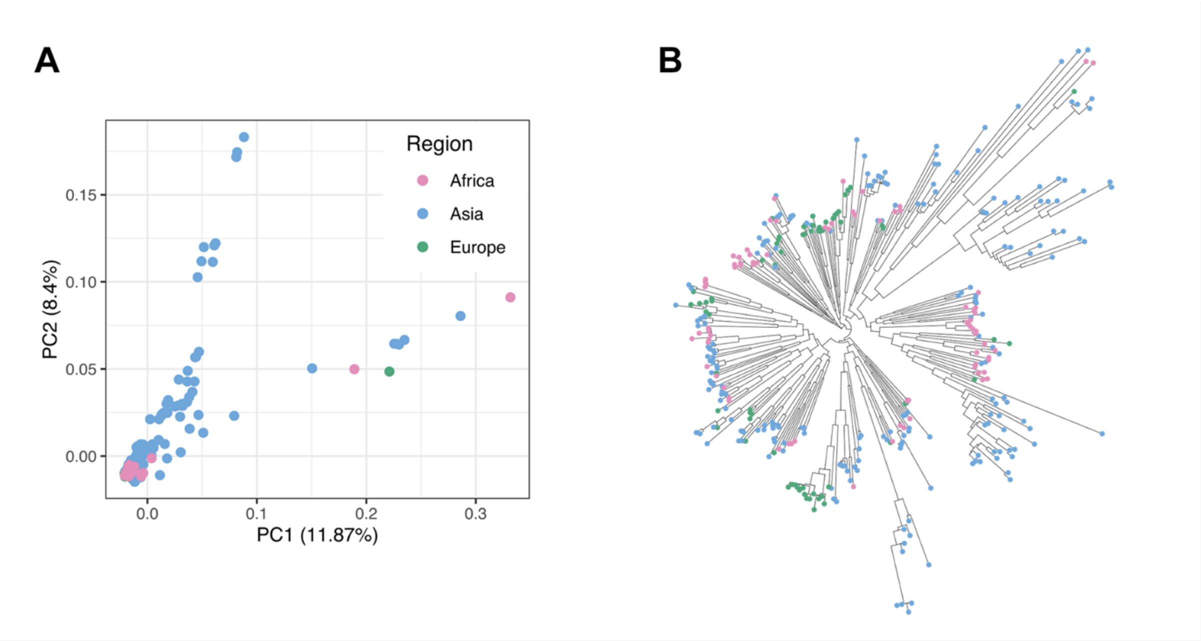

S4 Fig. Phylogenetic and population structure for *S. pneumoniae*'s total sample (including serotype NT). A. PCA computed in Plink v1.9 [1]. **B.** Maximum likelihood unrooted phylogenetic tree obtained using FastTree [2]. There are subclades of Asian lineages which belong exclusively to the PCA outlying serotypes NT (three subclades with isolates belonging mainly to GPSCs 28, 42, 60, 66, 118) and 19F (one subclade classified as GPSC-1), although both NT and 19F clades also include African and European lineages. The NT cluster comprise nonencapsulated isolates which were previously reported to have higher recombination rates generating significantly more diversity within this cluster [3]. Colour code corresponds to geographic region: Africa, pink; Asia, blue; Europe, green.

Supplementary References

[1] Purcell S, Neale B, Todd-Brown K, Thomas L, Ferreira MAR, Bender D, et al. PLINK: A tool set for whole-genome association and population-based linkage analyses. Am J Hum Genet. 2007;81(3):559-75. doi: 10.1086/519795. PubMed PMID: WOS:000249128200012.

[2] Price MN, Dehal PS, Arkin AP. FastTree 2-Approximately Maximum-Likelihood Trees for Large Alignments. Plos One. 2010;5(3). doi: 10.1371/journal.pone.0009490. PubMed PMID: WOS:000275328800002.

[3] Chewapreecha C, Harris SR, Croucher NJ, Turner C, Marttinen P, Cheng L, et al. Dense genomic sampling identifies highways of pneumococcal recombination. Nat Genet. 2014;46(3):305-+. doi: 10.1038/ng.2895. PubMed PMID: WOS:000332036700016.
