# Supplementary material for: Long-term evolution of Streptococcus mitis and Streptococcus pneumoniae leads to higher genetic diversity within rather than between human populations
Source: PLoS Genet. 2024 Jun 6;20(6):e1011317. doi: 10.1371/journal.pgen.1011317 (PMC11185502; doi:10.1371/journal.pgen.1011317)
Supplement: S5 Fig — The horizontal line corresponds to the expected number of genes with p-values<0.05, under a null uniform distribution of p-values. (PDF) [file pgen.1011317.s008.pdf]

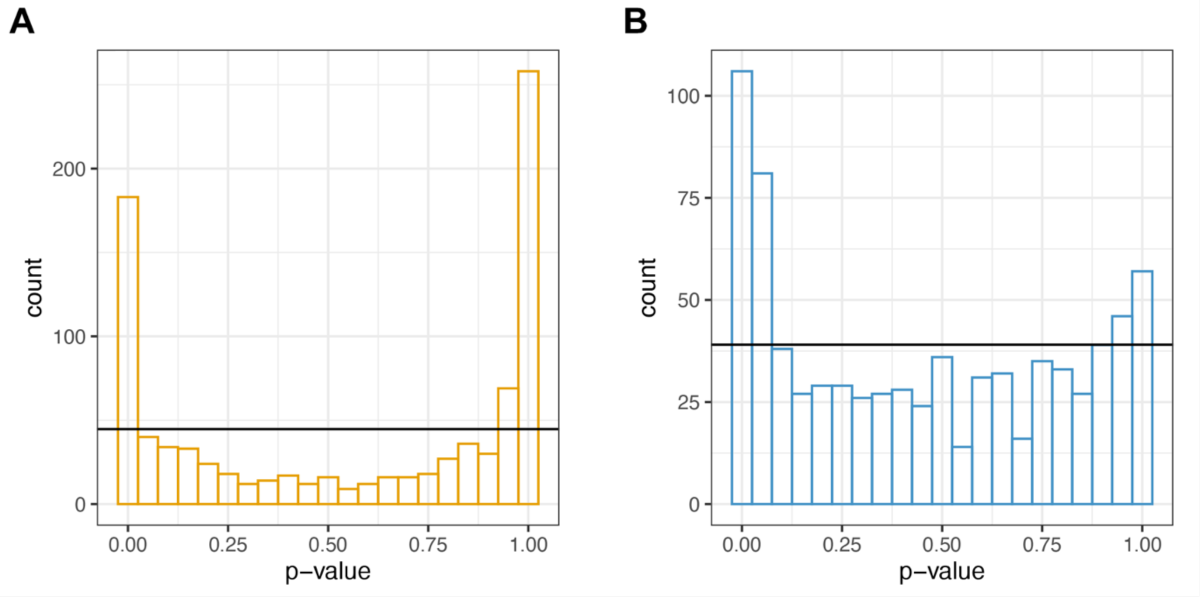

S5 Fig. Distribution of p-values for whether genes have a higher ratio of number of non-synonymous variants to number of synonymous variants than a random set of simulated ratios for *S. mitis* (**A**) and *S. pneumoniae* (**B**). The horizontal line corresponds to the expected number of genes with p-values<0.05, under a null uniform distribution of p-values.
