# Supplementary material for: Long-term evolution of Streptococcus mitis and Streptococcus pneumoniae leads to higher genetic diversity within rather than between human populations
Source: PLoS Genet. 2024 Jun 6;20(6):e1011317. doi: 10.1371/journal.pgen.1011317 (PMC11185502; doi:10.1371/journal.pgen.1011317)
Supplement: S6 Fig — (PDF) [file pgen.1011317.s009.pdf]

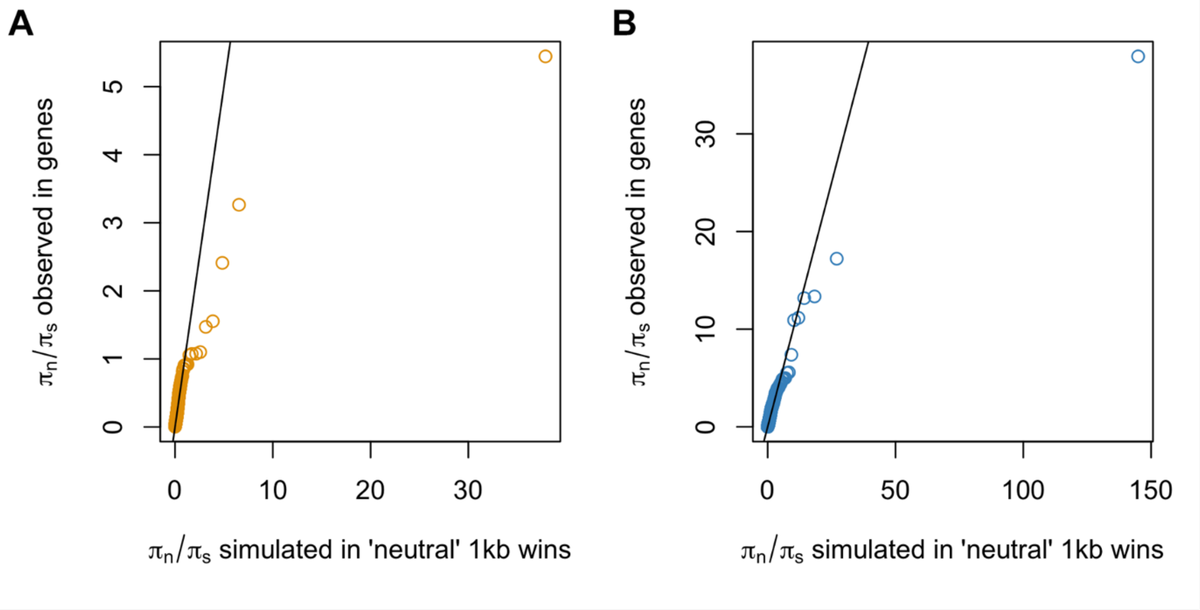

S6 Fig. QQplots of the null π_N/π_S distribution obtained from simulated 1Kb windows (wins) versus the observed π_N/π_S distributions in genes from *S. mitis* (**A**) and *S.pneumoniae* (**B**).
