# Supplementary material for: Long-term evolution of Streptococcus mitis and Streptococcus pneumoniae leads to higher genetic diversity within rather than between human populations
Source: PLoS Genet. 2024 Jun 6;20(6):e1011317. doi: 10.1371/journal.pgen.1011317 (PMC11185502; doi:10.1371/journal.pgen.1011317)
Supplement: S1 Table — Pangenome and core genome sizes units are number of genes. N, sample size. Regional (continental) random sample of unrelated isolates show means of 1000 random samples with size equal to that of the smaller regional sample size within each species. ‘All random sample of unrelated isolates’ in S. pneumoniae shows the mean of 1000 random samples with size equal to the observed S. mitis unrelated sample size. (PDF) [file pgen.1011317.s010.pdf]

S1 Table. Pangenome statistics for *S. pneumoniae* and *S. mitis*. Pangenome and core genome sizes units are number of genes. N, sample size. Regional (continental) random sample of unrelated isolates show means of 1000 random samples with size equal to that of the smaller regional sample size within each species. 'All random sample of unrelated isolates' in *S. pneumoniae* shows the mean of 1000 random samples with size equal to the observed *S. mitis* unrelated sample size.

Sample	N	Pangenome size	Core Genome size	Mean no. genes per sample	Median no. genes per sample
S. pneumoniae					
All	802	27006	902	2004	1999
African	222	6898	1067	2022	2010
Asian	480	8179	982	1994	1991
European	100	5188	1169	2016	2020
All unrelated	353	8811	934	2005	1995
African unrelated	78	6282	1075	2035	2014
Asian unrelated	207	7192	1013	1991	1985
European unrelated	68	5047	1175	2014	2020
African random sample of unrelated isolates	68	6215	1080	2038	2015
Asian random sample of unrelated isolates	68	5818	1101	1983	1982
All random sample of unrelated isolates	75	6064	1092	2005	1995
S. mitis					
All	119	12005	948	1876	1828
African	32	5520	1058	1774	1772
Asian	36	5288	1091	1841	1835
European	49	7004	992	1884	1864
All unrelated	75	9626	951	1832	1816
African unrelated	25	5495	1058	1775	1773
Asian unrelated	18	5242	1093	1847	1834
European unrelated	32	6897	993	1869	1862
African random sample of unrelated isolates	18	4764	1082	1761	1772
European random sample of unrelated isolates	18	5322	1071	1869	1864
