# Supplementary material for: Long-term evolution of Streptococcus mitis and Streptococcus pneumoniae leads to higher genetic diversity within rather than between human populations
Source: PLoS Genet. 2024 Jun 6;20(6):e1011317. doi: 10.1371/journal.pgen.1011317 (PMC11185502; doi:10.1371/journal.pgen.1011317)
Supplement: S2 Table — S. mitis, above diagonal; S. pneumoniae, below diagonal. (PDF) [file pgen.1011317.s011.pdf]

S2 Table. Between population divergence estimates (Hudson's F_{ST} +/- SE). *S. mitis,* above diagonal; *S. pneumoniae*, below diagonal.

	Africa	Asia	Europe
Africa		0.0684 +/- 0.0017	0.0495 +/- 0.0011
Asia	0.0306 +/- 0.0035		0.0107 +/- 0.0012
Europe	0.0406 +/- 0.0027	0.0464 +/- 0.0038	
