# Supplementary material for: Long-term evolution of Streptococcus mitis and Streptococcus pneumoniae leads to higher genetic diversity within rather than between human populations
Source: PLoS Genet. 2024 Jun 6;20(6):e1011317. doi: 10.1371/journal.pgen.1011317 (PMC11185502; doi:10.1371/journal.pgen.1011317)
Supplement: S3 Table — (PDF) [file pgen.1011317.s012.pdf]

S3 Table. Genome accession and geographical origin of *Streptococcus mitis* genomes.

Host	isolate	region	Biosample	Bioproject
B1015B24	B1015B24C5	Africa	SAMEA8000357	PRJEB42963
B1015G24	B1015G24C3	Africa	SAMEA8000358	PRJEB42963
B1015H24	B1015H24C1	Africa	SAMEA8000359	PRJEB42963
B1015H24	B1015H24C3	Africa	SAMEA8000360	PRJEB42963
B1015H24	B1015H24C4	Africa	SAMEA8000361	PRJEB42963
S1071B24	S1071B24C1	Africa	SAMEA8000362	PRJEB42963
S1071B24	S1071B24C2	Africa	SAMEA8000363	PRJEB42963
S1071G24	S1071G24C1	Africa	SAMEA8000364	PRJEB42963
S1071G24	S1071G24C2	Africa	SAMEA8000365	PRJEB42963
S1071G24	S1071G24C3	Africa	SAMEA8000366	PRJEB42963
S1071G24	S1071G24C4	Africa	SAMEA8000367	PRJEB42963
S1071G24	S1071G24C5	Africa	SAMEA8000368	PRJEB42963
S1072B24	S1072B24C4	Africa	SAMEA8000369	PRJEB42963
S1072B24	S1072B24C5	Africa	SAMEA8000370	PRJEB42963
S1072G24	S1072G24C4	Africa	SAMEA8000371	PRJEB42963
S1075M24	S1075M24C2	Africa	SAMEA8000372	PRJEB42963
S1075M24	S1075M24C3	Africa	SAMEA8000373	PRJEB42963
S1075M24	S1075M24C5	Africa	SAMEA8000374	PRJEB42963
S1086G24	S1086G24C1	Africa	SAMEA8000375	PRJEB42963
S1088B24	S1088B24C2	Africa	SAMEA8000376	PRJEB42963
S1088M24	S1088M24C1	Africa	SAMEA8000377	PRJEB42963
S1091B24	S1091B24C4	Africa	SAMEA8000378	PRJEB42963
S1092G24	S1092G24C1	Africa	SAMEA8000379	PRJEB42963
S1092G24	S1092G24C3	Africa	SAMEA8000380	PRJEB42963
S1092G24	S1092G24C4	Africa	SAMEA8000381	PRJEB42963
S1093G24	S1093G24C1	Africa	SAMEA8000382	PRJEB42963
S1093G24	S1093G24C2	Africa	SAMEA8000383	PRJEB42963
S1093G24	S1093G24C3	Africa	SAMEA8000384	PRJEB42963
S1096G24	S1096G24C5	Africa	SAMEA8000385	PRJEB42963
S1096M24	S1096M24C3	Africa	SAMEA8000386	PRJEB42963
S1096M24	S1096M24C5	Africa	SAMEA8000387	PRJEB42963
C1	C1C1	Asia	SAMN37997237	PRJNA1032561
C1	C1C2	Asia	SAMN37997238	PRJNA1032561
C1	C1C3	Asia	SAMN37997239	PRJNA1032561
C1	C1C4	Asia	SAMN37997240	PRJNA1032561
C1	C1C5	Asia	SAMN37997241	PRJNA1032561

C1	C1T1	Asia	SAMN37997242	PRJNA1032561
C1	C1T3	Asia	SAMN37997243	PRJNA1032561
C2	C2C1	Asia	SAMN37997244	PRJNA1032561
C2	C2C2	Asia	SAMN37997245	PRJNA1032561
C2	C2C3	Asia	SAMN37997246	PRJNA1032561
C2	C2C4	Asia	SAMN37997247	PRJNA1032561
C2	C2C5	Asia	SAMN37997248	PRJNA1032561
C2	C2C6	Asia	SAMN37997249	PRJNA1032561
C2	C2T1	Asia	SAMN37997250	PRJNA1032561
C2	C2T2	Asia	SAMN37997251	PRJNA1032561
C2	C2T3	Asia	SAMN37997252	PRJNA1032561
C2	C2T4	Asia	SAMN37997253	PRJNA1032561
C3	C3C1	Asia	SAMN37997254	PRJNA1032561
C3	C3C2	Asia	SAMN37997255	PRJNA1032561
C3	C3C3	Asia	SAMN37997256	PRJNA1032561
C3	C3C4	Asia	SAMN37997257	PRJNA1032561
C3	C3T1	Asia	SAMN37997258	PRJNA1032561
C4	C4C1	Asia	SAMN37997259	PRJNA1032561
C4	C4C2	Asia	SAMN37997260	PRJNA1032561
C4	C4C3	Asia	SAMN37997261	PRJNA1032561
C4	C4C4	Asia	SAMN37997262	PRJNA1032561
C5	C5C1	Asia	SAMN37997263	PRJNA1032561
C5	C5C2	Asia	SAMN37997264	PRJNA1032561
C5	C5C3	Asia	SAMN37997265	PRJNA1032561
C5	C5C4	Asia	SAMN37997266	PRJNA1032561
C5	C5C5	Asia	SAMN37997267	PRJNA1032561
C5	C5T1	Asia	SAMN37997268	PRJNA1032561
C5	C5T2	Asia	SAMN37997269	PRJNA1032561
C5	C5T3	Asia	SAMN37997270	PRJNA1032561
C5	C5T4	Asia	SAMN37997271	PRJNA1032561
C5	C5T5	Asia	SAMN37997272	PRJNA1032561
SK1126	SK1126	Asia	SAMN02836935	PRJNA242574
B1	B1C1	Europe	SAMN37997273	PRJNA1032561
B1	B1C2	Europe	SAMN37997274	PRJNA1032561
B1	B1C3	Europe	SAMN37997275	PRJNA1032561

B1	B1C4	Europe	SAMN37997276	PRJNA1032561
B1	B1C5	Europe	SAMN37997277	PRJNA1032561
B1	B1T1	Europe	SAMN37997278	PRJNA1032561
B1	B1T2	Europe	SAMN37997279	PRJNA1032561
B2	B2C1	Europe	SAMN37997280	PRJNA1032561
B2	B2C2	Europe	SAMN37997281	PRJNA1032561
B2	B2C3	Europe	SAMN37997282	PRJNA1032561
B2	B2C4	Europe	SAMN37997283	PRJNA1032561
B2	B2T1	Europe	SAMN37997284	PRJNA1032561
B2	B2T2	Europe	SAMN37997285	PRJNA1032561
B2	B2T3	Europe	SAMN37997286	PRJNA1032561
B2	B2T4	Europe	SAMN37997287	PRJNA1032561
B3	B3C1	Europe	SAMN37997288	PRJNA1032561
B3	B3C3	Europe	SAMN37997289	PRJNA1032561
B3	B3C4	Europe	SAMN37997290	PRJNA1032561
B3	B3T1	Europe	SAMN37997291	PRJNA1032561
B3	B3T2	Europe	SAMN37997292	PRJNA1032561
B4	B4C1	Europe	SAMN37997293	PRJNA1032561
B4	B4C2	Europe	SAMN37997294	PRJNA1032561
B4	B4C3	Europe	SAMN37997295	PRJNA1032561
B4	B4C4	Europe	SAMN37997296	PRJNA1032561
B4	B4T1	Europe	SAMN37997297	PRJNA1032561
B5	B5C1	Europe	SAMN37997298	PRJNA1032561
B5	B5C2	Europe	SAMN37997299	PRJNA1032561
B5	B5C3	Europe	SAMN37997300	PRJNA1032561
B5	B5C4	Europe	SAMN37997301	PRJNA1032561
B5	B5C5	Europe	SAMN37997302	PRJNA1032561
B5	B5T1	Europe	SAMN37997303	PRJNA1032561
B5	B5T2	Europe	SAMN37997304	PRJNA1032561
SK1073	SK1073	Europe	SAMN00621702	PRJNA66111
SK1080	SK1080	Europe	SAMN00621705	PRJNA66113
SK137	SK137	Europe	SAMN03334900	PRJNA274768
SK145	SK145	Europe	SAMN03334902	PRJNA274768
SK271	SK271	Europe	SAMN02698681	PRJNA242568
SK321	SK321	Europe	SAMN00001433	PRJNA33353
SK564	SK564	Europe	SAMN00001434	PRJNA33355

SK569	SK569	Europe	SAMN00621701	PRJNA67187
SK575	SK575	Europe	SAMN00761799	PRJNA75135
SK578	SK578	Europe	SAMN02836940	PRJNA242567
SK579	SK579	Europe	SAMN00761835	PRJNA75157
SK597	SK597	Europe	SAMN00001435	PRJNA33357
SK608	SK608	Europe	SAMN02836941	PRJNA242555
SK616	SK616	Europe	SAMN00761793	PRJNA75129
SK629	SK629	Europe	SAMN02836936	PRJNA242570
SK637	SK637	Europe	SAMN02836939	PRJNA242571
SK642	SK642	Europe	SAMN02836938	PRJNA242572
SK667	SK667	Europe	SAMN02836937	PRJNA242573
